# Acid-Responsive Decomposable Nanomedicine Based on Zeolitic Imidazolate Frameworks for Near-Infrared Fluorescence Imaging/Chemotherapy Combined Tumor Theranostics

**DOI:** 10.3390/pharmaceutics16060823

**Published:** 2024-06-18

**Authors:** Heze Guo, Vincent Mukwaya, Daikun Wu, Shuhan Xiong, Hongjing Dou

**Affiliations:** The State Key Laboratory of Metal Matrix Composites, School of Materials Science and Engineering, Shanghai Jiao Tong University, Shanghai 200240, China

**Keywords:** ZIF-8 nanoparticles, acid-responsiveness, NIRF imaging, chemotherapy, tumor theranostics

## Abstract

Zeolitic imidazolate framework-8 (ZIF-8) nanoparticles (NPs) are gaining traction in tumor theranostics for their effectiveness in encapsulating both imaging agents and therapeutic drugs. While typically, similar hydrophilic molecules are encapsulated in either pure aqueous or organic environments, few studies have explored co-encapsulation of chemotherapeutic drugs and imaging agents with varying hydrophilicity and, consequently, constructed multifunctional ZIF-8 composite NPs for acid-responsive, near-infrared fluorescence imaging/chemotherapy combined tumor theranostics. Here, we present a one-pot method for the synthesis of uniform Cy5.5&DOX@ZIF-8 nanoparticles in mixed solvents, efficiently achieving simultaneous encapsulation of hydrophilic doxorubicin (DOX) and hydrophobic Cyanine-5.5 (Cy5.5). Surface decoration with dextran (Dex) enhanced colloidal stability and biocompatibility. The method significantly facilitated co-loading of Cy5.5 dyes and DOX drugs, endowing the composite NPs with notable fluorescent imaging capabilities and pH-responsive chemotherapy capacities. In vivo near-infrared fluorescence (NIRF) imaging in A549 tumor-bearing mice demonstrated significant accumulation of Cy5.5 at tumor sites due to enhanced permeability and retention (EPR) effects, with fluorescence intensities approximately 48-fold higher than free Cy5.5. Enhanced therapeutic efficiency was observed in composite NPs compared to free DOX, validating tumor-targeted capability. These findings suggest ZIF-8-based nanomedicines as promising platforms for multifunctional tumor theranostics.

## 1. Introduction

Metal-organic frameworks (MOFs), known as porous coordination networks, are well-defined crystalline materials that are composed of metal ions and organic ligands [1,2,3]. The MOFs possess numerous fascinating attributes, such as large surface areas, high porosity, tunable structures, versatile functionality, and so forth [4,5,6]. These properties ensure that MOFs are ideal materials for applications in the areas of catalysis [7], gas storage/separation [8], energy storage [9], sensing [10], and drug delivery [4,11]. As a widely and deeply investigated MOF, zeolitic imidazolate framework-8 (ZIF-8), constructed from zinc ions (Zn^2+^) and 2-methylimidazolate (2-mim), has demonstrated remarkable applications in the field of biomedicine due to the biocompatible synthetic process, facile modification, favorable biocompatibility, high drug payload, and pH sensitivity [12,13,14]. With the open porous structures, accessible organic active sites, and good stability of ZIF-8 nanoparticles (NPs), various hydrophilic or hydrophobic cargoes, including imaging agents [15], Photosensitizers [16] and chemotherapeutic drugs [15,17], can be efficiently loaded into ZIF-8 NPs. In addition, the ZIF-8 NPs not only provide innovative avenues for promoting the immobilization of the abovementioned bio-macromolecules, but also offer efficacious protection for the internal payloads against external environments [18,19]. Moreover, it is worth noting that ZIF-8 is highly stable under normal physiological conditions, but it undergoes rapid degradation in acidic microenvironments due to the protonation of 2-mim, which consequently destroys the architecture of ZIF-8 [14,20]. Based on this pH-responsive behavior, ZIF-8 NPs loaded with multifunctional molecules have captured widespread attention as potential drug delivery systems, offering pH-responsive release for tumor imaging and therapy [20,21,22].

ZIF-8 particles can be fabricated by various strategies, such as solvothermal, microwave, sonochemical, mechanochemical, dry-gel conversion, microfluidic, and electrochemical methods [23,24,25]. Among them, the solvothermal method is the most widely adopted approach to obtain ZIF-8 NPs and their composite NPs loaded with functional molecules in organic solvents or water [15,26]. Moreover, ZIF-8 NPs, serving as nanocarriers encapsulating functional molecules, are usually prepared via two-step or one-pot approaches [17,26,27]. Specifically, the former approach separates the construction of the ZIF-8 structure and the encapsulation of functional molecules as two distinct processes, whereas the latter strategy integrates the two processes synchronously, resulting in more abundant encapsulation of functional molecules [17,26]. During synthesis via the one-pot method, the immobilization of functional molecules is achieved in either aqueous or organic solvents, depending on the solubility of the molecules [28,29,30,31,32]. Namely, the encapsulation of hydrophilic molecules greatly dissolved in water is normally conducted in aqueous solution [29,30], while the loading of hydrophobic molecules that are poorly dispersed in water is performed in organic solvents [31,32]. As reported, dimethyl sulfoxide/water (DMSO/H_2_O) mixed solvents can be used to simultaneously immobilize hydrophilic photo-thermal molecules and hydrophobic imaging agents into ZIF-8 NPs for tumor photothermal therapy and fluorescence imaging [33,34]. However, there is still few specific studies that allow for the sufficient co-encapsulation of chemotherapeutic drugs and imaging agents with varying hydrophilicity, resulting in the construction of multifunctional ZIF-8 composite NPs via the one-pot method for acid-responsive, near-infrared fluorescence imaging/chemotherapy combination theranostics.

**Scheme 1:**
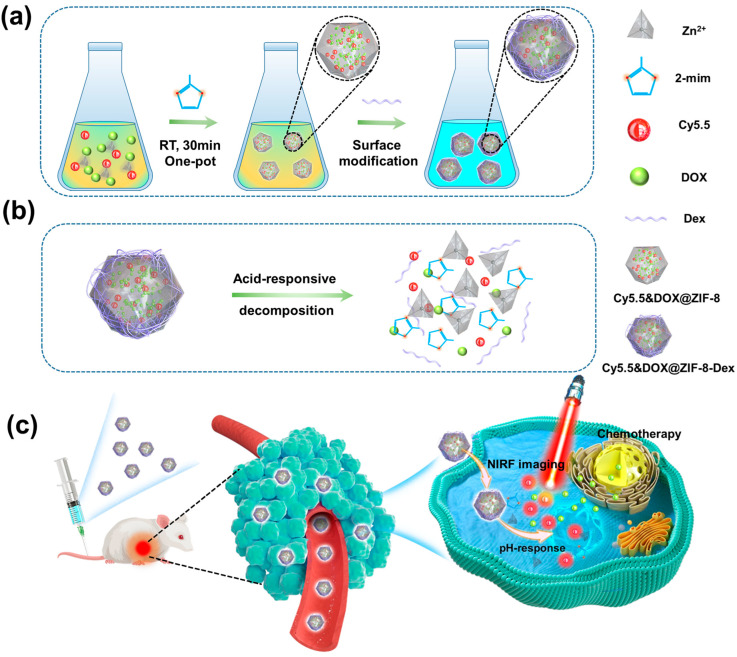
(**a**) Schematic illustration depicting the construction of Cy5.5&DOX@ZIF-8-Dex composite NPs that were simultaneously loaded with the imaging agent Cy5.5 and drug DOX, and the subsequent decoration of dextran on the surfaces of these NPs. (**b**) Schematic illustration depicting the decomposition of Cy5.5&DOX@ZIF-8 NPs and the releasing of loaded cargoes under an acid environment. (**c**) Illustration depicting their application as a theranostic delivery system for tumor NIRF imaging and pH-responsive chemotherapy.

Among the numerous imaging agents that exist, Cyanine-5.5 (Cy5.5) is a hydrophobic NIRF imaging agent possessing numerous desirable attributes, such as non-invasiveness, high flexibility, real-time imaging, and remarkable sensitivity, thus demonstrating potential applications in tumor diagnosis [35,36]. On the other hand, the hydrophilic molecule, doxorubicin (DOX) hydrochloride, acts as a chemotherapeutic drug for anticancer chemotherapy and is one of the most widely used drugs in tumor therapy [37,38]. Furthermore, the hydrophobic Cy5.5 dyes are efficiently dissolved in DMSO, while the hydrophilic DOX molecules are easily dispersed in water. In addition, the direct use of free Cy5.5 and DOX for tumor theranostics may result in insufficient accumulation at tumor sites due to the native aggregation of hydrophobic molecules and the rapid clearance from the human body by the circulatory system [39,40]. As described herein, we have developed a facile approach to prepare Cy5.5&DOX@ZIF-8 composite NPs in DMSO/H_2_O mixed solvents for near-infrared fluorescence (NIRF) imaging and pH-responsive chemotherapy of tumors, which were surface-modified with amino-dextran [33,41,42,43] to further improve the colloidal stability and biocompatibility of the as-prepared NPs (Scheme 1a). The construction of ZIF-8 NPs and the co-loading of functional molecules could be concurrently achieved in the mixed solvent system, where the hydrophobic Cy5.5 dyes were dissolved in DMSO, and the hydrophilic DOX molecules were dispersed in water. The as-synthesized Cy5.5&DOX@ZIF-8-Dex NPs, with uniform size, regular morphology, and high colloidal stability, could prevent the aggregation of Cy5.5, promote the tumor-targeting capability due to the enhanced permeability and retention effect (EPR) [39,40,44], and consequently enhance the effect of tumor theranostics. Most importantly, the unique pH sensitivity of the ZIF-8 NPs, which can be specifically activated by the acidic microenvironments encountered in tumors, enabled the co-loaded ZIF-8 composite NPs to responsively release drugs, thus achieving minimal side effects and optimal theranostic effects (Scheme 1b,c). Therefore, this study may further widen the scope of synthetic strategies and applications for ZIF-8 composite NPs capable of simultaneously encapsulating functional molecules for acid-responsive, near-infrared fluorescence imaging/chemotherapy combined theranostics.

## 2. Materials and Methods

### 2.1. Materials

The 2-methylimidazole (analytical grade) was purchased from TCI Shanghai Co., Ltd., Shanghai, China. Amino-dextran (*M*_w_ ca. 40 kDa) was provided by Thermo Fisher Scientific Co., Ltd., Waltham, MA, USA. Zinc nitrate hexahydrate of analytical grade was purchased from Shanghai Adamas Reagent Co., Ltd., Shanghai, China. Dimethyl sulfoxide (DMSO, analytical grade) was purchased from Sinopharm Chemical Reagent Co., Ltd., Shanghai, China. Cyanine-5.5-NHS (Cy5.5-NHS, nearly insoluble in water and greatly soluble in DMSO) was supplied by Lumiprobe Corporation, Hunt Valley, MD, USA. Doxorubicin hydrochloride (DOX, highly soluble in aqueous solution) was provided by Beijing InnoChem Scientific Co., Ltd., Beijing, China. Fetal bovine serum (FBS) was supplied by Biological Industries, Cromwell, CT, USA. Trypsin-EDTA solution and Roswell Park Memorial Institute-1640 (RPMI-1640) were purchased from Sigma-Aldrich, St. Louis, MI, USA. Phosphate-buffered saline (PBS), Cell Counting Kit-8 (CCK-8), and 4’,6-diamidino-*2*-phenylindole (DAPI) were purchased from Shanghai Beyotime Biotechnology Co., Ltd., Shanghai, China. Paraformaldehyde fixative (4 wt%) was provided by NanJing KeyGen Biotechnology Co., Ltd., Nanjing, China. Human umbilical vein endothelial cell (HUVEC) and human non-small-cell lung carcinoma (A549) were purchased from the Animal Institute of the Chinese Academy of Science, Shanghai, China. The nude mice were offered by Shanghai SLAC Laboratory Animal Co., Ltd., Shanghai, China.

### 2.2. Preparation of the Cy5.5&DOX@ZIF-8 NPs

The composite Cy5.5&DOX@ZIF-8 NPs were synthesized in DMSO/H_2_O solvent mixtures [33,34]. For the typical preparation of the multifunctional composite NPs, stock aqueous solutions of Zn(NO_3_)_2_·6H_2_O (20 wt%) and 2-methylimidazole (2-mim, 20 wt%) were prepared beforehand. Initially, 2880 μL of DMSO and 920 μL of H_2_O were added into 400 μL of an aqueous Zn(NO_3_)_2_·6H_2_O solution. The mixed solution was subsequently stirred at a speed of 1500 rpm for 10 min. Subsequently, a 200 μL DMSO solution of Cy5.5 (0.5 mg/mL) and 500 μL of an aqueous solution of DOX (2 mg/mL) were added dropwise to the mixtures under stirring. After the mixed solution had been vibrated for 30 min, 4 mL of an aqueous 2-mim solution was subsequently added to the above mixtures. At this moment, the proportion of DMSO in the mixed solvents was set as 35 vol% and the Zn^2+^-to-2-mim molar ratio was 1:36, which were the suitable parameters to prepare uniform ZIF-8 nanoparticles [34]. After they had been stirred at a rate of 1200 rpm for 30 min, the Cy5.5&DOX@ZIF-8 NPs were finally collected via centrifugation, during which the as-prepared NPs were washed three times with water to completely remove both the DMSO solvent and the unreacted reactants. All of these procedures were conducted at room temperature. Samples for dynamic light scattering (DLS) measurements and transmission electron microscopy (TEM) observations were prepared by redispersing Cy5.5&DOX@ZIF-8 NPs into deionized water. Additionally, the products that would be employed for other characterizations were dried under a vacuum at −80 °C. Furthermore, the Cy5.5@ZIF-8 and DOX@ZIF-8 NPs were prepared in a similar manner for comparison. In addition, the same volume fraction of DMSO in the mixed solution (35 vol%) and the Zn^2+^-to-2-mim molar ratio (1:36), as mentioned earlier, were also used in this case.

### 2.3. Surface Modification of the Cy5.5&DOX@ZIF-8-Dex NPs

Amino-dextran was incorporated onto the surfaces of the composite NPs via coordination bonds to yield dextran-modified Cy5.5&DOX@ZIF-8-Dex NPs [33,41,45]. In a typical procedure, the as-prepared Cy5.5&DOX@ZIF-8 NPs (2 mg/mL) were dispersed into water and were subsequently sonicated for 10 min to further enhance the dispersity of these NPs. Following this process, 500 μL of dextran aqueous solution (4 mg/mL) was added into 3 mL of the aforementioned solution and subsequently stirred at a rate of 1000 rpm for 12 h. The dextran-capped Cy5.5&DOX@ZIF-8-Dex NPs could then be obtained in the form of a precipitate by means of centrifugation (12,000 rpm, 15 min). The products were subsequently washed several times in deionized water to completely wash away the un-adsorbed dextran. Samples of the NPs were redispersed into deionized water prior to TEM and DLS characterizations. Alternatively, the multifunctional composite ZIF-8 NPs were dried at −80 °C under a vacuum prior to analysis via other techniques.

### 2.4. Characterization

A DLS system (Malvern Zetasizer Nano ZS90, Malvern, Worcestershire, UK) was employed to measure the hydrodynamic diameters and the zeta potentials of the composite NPs. The morphologies of the various composite NPs were characterized by TEM (JEM-2100, JEOL, Tokyo, Japan), with an accelerating voltage of 200 kV. Meanwhile, the corresponding size distributions based on 100 particles were evaluated with the aid of ImageJ software (ImageJ 1.51k; Java 1.6.0_24). Fluorescence spectra of Cy5.5&DOX@ZIF-8-Dex NPs were acquired at various concentrations with a fluorescence spectrophotometer (RF-5301, Shimadzu, Tokyo, Japan). The ultraviolet and visible (UV-Vis) absorption spectra of all the composite NPs that had been loaded with various functional molecules were characterized with the use of a UV-Vis spectrophotometer (UV-2450, Shimadzu, Tokyo, Japan). Meanwhile, the FT-IR spectroscopy measurements of the as-prepared NPs were conducted over the range of 400–4000 cm^−1^ utilizing a Nicolet 6700 spectrometer (Thermo Fisher Scientific Co., Ltd., Waltham, MA, USA), with KBr pellets as the sample matrix. A gas sorption analyzer (Autosorb-IQ, Quantachrome Instruments, Boynton Beach, FL, USA) was employed to measure the nitrogen sorption isotherms of the composite NPs at 77K. An X-ray diffractometer (D8 Advance, Bruker Corporation, Karlsruhe, Germany) utilizing CuKα radiation, which was operated at a scanning rate of 5° min^−1^, was employed to record the X-ray diffraction (XRD) spectra of the samples over the 2θ range of 5–30°.

### 2.5. In Vitro Release of Cy5.5 and DOX from pH-Sensitive Cy5.5&DOX@ZIF-8-Dex NPs

Here, 5 mg of Cy5.5&DOX@ZIF-8-Dex NPs were dispersed in 5 mL of 20 mM PBS buffer solution at various pH levels (7.4, 5.8, and 4.8). Subsequently, the as-prepared solution was transferred into a dialysis bag (Kw = 3500 Da) along with 15 mL of the corresponding buffer solution. At predetermined time intervals, 500 μL of external dialysate solution was withdrawn into a quartz cuvette for Cy5.5 and DOX concentration quantification. The Cy5.5 release profile was calculated at 675 nm and the DOX release profile was determined at 488 nm using a UV spectrophotometer. Furthermore, the in vitro release of Cy5.5 from Cy5.5@ZIF-8-Dex and the DOX release profile from DOX@ZIF-8-Dex were performed in a similar way.

### 2.6. Cell Culture Experiments

The cellular uptake and anticancer effect of the Cy5.5@ZIF-8-Dex, DOX@ZIF-8-Dex, and Cy5.5&DOX@ZIF-8-Dex NPs on two types of cancer cells were evaluated, namely, human umbilical vein endothelial cells (HUVEC) and human non-small-cell lung carcinoma cells (A549). These cells were, respectively, incubated in RPMI-1640 culture media consisting of fetal bovine serum (FBS, 10 vol%), streptomycin (100 U/mL), and penicillin (100 U/mL). Subsequently, these cell samples were kept at 37 °C for 48 h in a humidified atmosphere of 5% CO_2_.

### 2.7. In Vitro Cytotoxicity

CCK-8 assay tests were first conducted to evaluate the in vitro cell viability of cells treated with the Cy5.5&DOX@ZIF-8-Dex NPs. HUVEC cells and A549 tumor cells were introduced into the 96-well plates at a density of 10^4^ cells per well, respectively. After these cells had been cultivated overnight, they were, respectively, incubated for 24 h and 48 h with the fresh media consisting of Cy5.5&DOX@ZIF-8-Dex NPs at different concentrations (10, 25, 50, 100, 200, and 400 μg/mL). The culture media were subsequently removed, and the cells were incubated with CCK-8 solution for 2 h prior to the cell viability measurements. The absorbance (*A*) at 490 nm was measured with the use of a microplate reader. The cytotoxicity measurements of all the samples were conducted in triplicate and the corresponding cell viability rates were thus calculated according to Equation (1):

(1) $$\mathrm{Cell}\;\mathrm{viability}\;(\%)=\frac{A_{1}-A_{3}}{A_{2}-A_{3}}\;\times \;100$$

where *A*_1_ denotes the absorbance of cells, samples, and CCK-8 solution, *A*_2_ represents the absorbance of the cells and the CCK-8 solution, and *A*_3_ is the absorbance of the CCK-8 solution. Furthermore, the CCK-8 assay tests on both Cy5.5@ZIF-8-Dex and DOX@ZIF-8-Dex NPs were performed via the same method.

### 2.8. Evaluation of the Cellular Uptake

In order to confirm the cellular uptake of Cy5.5&DOX@ZIF-8-Dex NPs, the A549 cells were first cultured at a concentration of 10^5^ cells/mL in the special plate of CLSM for 12 h. Subsequently, the media were removed, and the cells were then replaced with fresh culture media consisting of Cy5.5@ZIF-8-Dex, DOX@ZIF-8-Dex, and Cy5.5&DOX@ZIF-8-Dex NPs, respectively. After the cells were further cultivated for 3 h, they were washed three times with PBS buffer to wash away the residual materials. Subsequently, 4 wt% paraformaldehyde fixative was utilized to immobilize the A549 cells for 30 min. Lastly, the cells were rinsed with PBS buffer and were subsequently stained with DAPI for 20 min. Confocal laser scanning microscopy (CLSM) imaging was employed to record the fluorescent images of each sample. In order to further quantify the cellular uptake of Cy5.5&DOX@ZIF-8-Dex, cell sorting analysis was performed to evaluate the fluorescent intensities of stained cells. The cells were incubated for 3 h with culture media consisting of Cy5.5@ZIF-8-Dex, DOX@ZIF-8-Dex, and Cy5.5&DOX@ZIF-8-Dex NPs, respectively. These A549 cells were collected via trypsinization, then washed twice with PBS buffer solution, and finally suspended in PBS buffer prior to flow cytometry measurements.

### 2.9. Animal Preparation and Tumor Models

Female BALB/c nude mice weighing ~20 g (6–8 weeks in age) were provided and further maintained under specific pathogen-free conditions by the Shanghai SLAC Laboratory Animal Center (Shanghai, China). All animal experiments were performed under the guidance of the National Institutes of Health Guidelines for the Care and Use of Laboratory Animals. Prior to the experiments, the mice were completely anesthetized via administration with isoflurane.

The A549 tumor cells within the logarithmic growth phase were dispersed in PBS solution at a cell density of 5.0 × 10^7^ cells/mL. The tumor models were obtained by subcutaneously injecting 0.1 mL of these cell suspensions into each mouse at the anterior armpit. Tumor growth was monitored based on measurements of the tumor diameters, which were determined with the use of a Vernier caliper. In addition, the tumor volumes were calculated via Equation (2):

Tumor volume = (*L*_M_ × *L*_m_^2^)/2 (2)

where *L*_m_ (mm) and *L*_M_ (mm), respectively, denote the minimum and maximum measured lengths of the tumor.

### 2.10. In Vivo NIRF Imaging Experiments

The distribution of the Cy5.5&DOX@ZIF-8-Dex NPs within the mice was determined via in vivo near-infrared fluorescence (NIRF) imaging with the use of an IVIS Lumina Ⅱ in vivo imaging system. The A549 tumor-bearing mice were completely anesthetized under isoflurane exposure prior to the in vivo NIRF imaging. When the tumor volumes reached approximately 150 mm^3^, Cy5.5&DOX@ZIF-8-dex (100 μL and 2 mg/mL) was administered to the mice via tail vein injection. Meanwhile, the other mice were, respectively, injected with PBS buffer (100 μL) and free Cy5.5 (100 μL and 70 μg/mL) as a blank group and control group, which guaranteed the equivalent amount of Cy5.5 within the mice. At predetermined times post-injection (0.5, 1, 2, 4, 8, 12, 16, 24, and 48 h), fluorescent images were acquired by means of the in vivo imaging system. The corresponding mice were sacrificed after the in vivo NIRF imaging was performed to evaluate the distributions of Cy5.5 in the tissues and tumors of the A549 tumor-bearing mice. Along with the tumors, various individual organs, including the lung, heart, spleen, kidneys, liver, and muscle, were carefully dissected, and their fluorescent images and intensities were measured with the use of the in vivo imaging system.

### 2.11. Evaluation of In Vivo Chemotherapy

The A549 tumor-bearing mice were randomly divided into four groups (*n* = 6) when the tumor volumes reached approximately 150 mm^3^. These four groups were then separately administered with 100 μL of PBS buffer, free DOX (0.28 mg/mL), DOX@ZIF-8-Dex (1.75 mg/mL), or Cy5.5&DOX@ZIF-8-Dex (2 mg/mL) via tail injection every 3 days for 2 weeks, whereby the same amount of DOX was administered to each group (except for the group treated with PBS buffer). Subsequently, the tumor sizes and body weights were monitored every two days over a three-week period. The aforementioned Equation (2) was used to calculate the tumor volumes present in all of the groups. These mice were eventually euthanized in order to harvest the tumors so that accurate tumor volumes could be determined according to Equation (3):

Tumor volume = 4/3 × π × L/2 × W/2 × H/2(3)

where *L*, *W*, and *H*, respectively, denote the length, width, and height of the harvested tumor.

### 2.12. Histopathological Analysis

Histopathological analysis of tissue sections stained with hematoxylin and eosin staining (H&E staining) agents was performed to further assess the biosafety of the Cy5.5&DOX@ZIF-8-Dex NPs. The A549 tumor-bearing mice were sacrificed, and the major organs of the above-described four groups (including the liver, spleen, heart, lung, and kidneys) were harvested, subsequently fixed in 4 wt% formalin, and eventually embedded in paraffin. Subsequently, these organs were cut into slices and stained with hematoxylin and eosin (H&E) to enable histopathological characterization via optical microscopy.

### 2.13. Statistical Analysis

Quantitative results were shown as mean values ± standard deviation (SD), resulting from at least three independent measurements. Individual groups were compared by utilizing Student’s *t*-test. The difference between control and experimental groups was determined to be statistically significant when the probability (*p*) value was lower than 0.05. The statistical significance was further set at *p*-values of (*) *p* < 0.05, (**) *p* < 0.01, and (***) *p* < 0.001.

## 3. Results and Discussion

### 3.1. Preparation of the Cy5.5&DOX@ZIF-8-Dex NPs

Considering the remarkable solubility of hydrophobic Cy5.5 in DMSO and dispersity of hydrophilic DOX in water, we envisioned that the use of DMSO/H_2_O solvent mixtures would provide an effective approach to construct uniform Cy5.5&DOX@ZIF-8 NPs, which could be efficiently and simultaneously co-loaded with the NIRF imaging agent Cy5.5 and the chemotherapeutic drug DOX [33,34]. To validate the feasibility of the DMSO/H_2_O mixed system and the superiority of the one-pot approach [26,32], Cy5.5@ZIF-8 and DOX@ZIF-8 NPs were, respectively, prepared in the hybrid solvents via one-pot or two-step methods prior to the preparation of Cy5.5&DOX@ZIF-8 NPs. The loading capacities of Cy5.5@ZIF-8 and DOX@ZIF-8 NPs, as shown in Figures S1 and S2a,b in the Supplementary Materials, were evaluated based on their UV-Vis spectra and their corresponding calibration curves. The DOX-loading percentage (18.8 ± 2.1%) observed in the DOX@ZIF-8 NPs constructed via the one-pot approach was found to be approximately eight times greater than that achieved in the DOX@ZIF-8 NPs prepared via the two-step method. Meanwhile, the Cy5.5-loading percentage (9.7 ± 1.3%) exhibited by Cy5.5@ZIF-8 NPs obtained via the one-pot approach also showed an impressive enhancement compared with that of the two-step method. Based on the characteristic peaks of free Cy5.5 and DOX (675 and 488 nm, respectively), the presence of the corresponding absorption peaks in the UV-Vis spectra of the composite ZIF-8 NPs confirmed that the Cy5.5 and DOX had been successfully immobilized in these NPs. Moreover, both of these characteristic peaks exhibited red shifts, which thus confirmed that complexation had occurred between the loaded molecules and the precursors of ZIF-8 [26]. These results demonstrated that considerable loading was achieved via the one-pot approach, which was subsequently employed to prepare multifunctional ZIF-8 NPs that were co-loaded with hydrophobic Cy5.5 and hydrophilic DOX.

**Figure 1:**
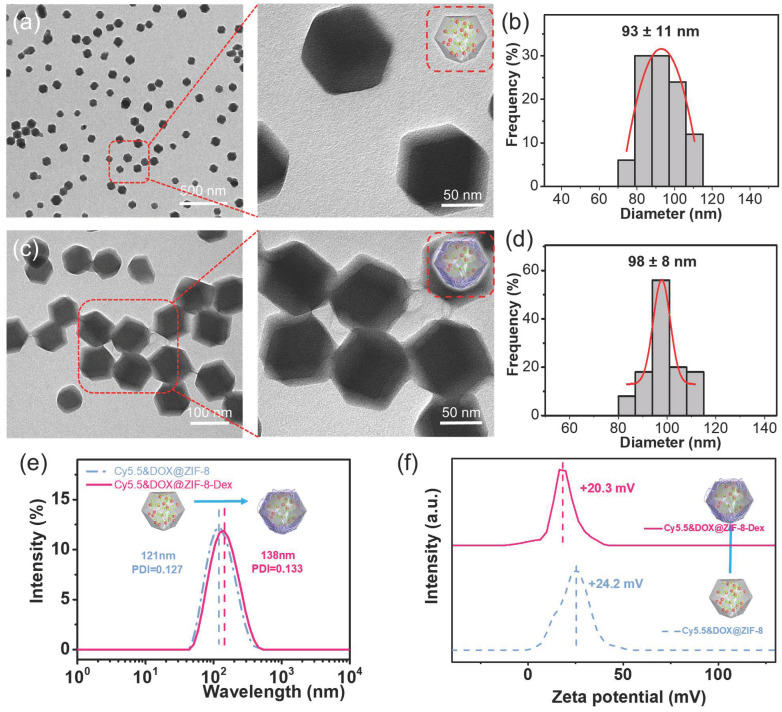
TEM images of: (**a**) Cy5.5&DOX@ZIF-8 composite NPs (**c**) and dextran-modified Cy5.5&DOX@ZIF-8-Dex composite NPs. Size distributions of: (**b**) Cy5.5&DOX@ZIF-8 composite NPs and (**d**) dextran-modified Cy5.5&DOX@ZIF-8-Dex composite NPs. (**e**) DLS curves of Cy5.5&DOX@ZIF-8 and dextran-modified Cy5.5&DOX@ZIF-8-Dex NPs. (**f**) Zeta potentials of Cy5.5&DOX@ZIF-8 and dextran-modified Cy5.5&DOX@ZIF-8-Dex NPs.

Transmission electron microscopy (TEM) and dynamic light scattering (DLS) characterization were conducted to evaluate the sizes and morphologies of the composite NPs, as shown in Figure 1. The TEM images demonstrated that the Cy5.5&DOX@ZIF-8 NPs exhibited regular hexagonal morphologies with regular shapes and uniform sizes, both before and after the incorporation of dextran on their surfaces. In addition, the size distribution revealed that the diameter of the dextran-decorated Cy5.5&DOX@ZIF-8-Dex NPs (98 ± 8 nm, Figure 1d) was slightly larger than that of the corresponding Cy5.5&DOX@ZIF-8 NPs that had not undergone dextran modification (93 ± 11 nm, Figure 1b). Moreover, the hydrodynamic diameter of the Cy5.5&DOX@ZIF-8 NPs determined via the DLS curves was approximately 120 nm, while that of Cy5.5&DOX@ZIF-8-Dex was around 140 nm (Figure 1e). Both of these diameters were larger than the abovementioned diameters that were obtained via the TEM observation. This discrepancy is due to the fact that the DLS measurements represented the hydrodynamic diameters of NPs, while TEM observation showed the diameters of NPs after they had been dried. Meanwhile, the larger diameters for Cy5.5&DOX@ZIF-8-Dex in comparison with Cy5.5&DOX@ZIF-8 (as observed via both TEM and DLS characterization) can be ascribed to the incorporation of dextran on the surfaces of the composite ZIF-8 NPs. The zeta potential of the composite NPs decreased by a modest degree after surface decoration (Figure 1f) due to the incorporation of dextran, which covered the surfaces of the Cy5.5&DOX@ZIF-8-Dex NPs via non-covalent interactions. Similarly, the TEM and DLS characterization of pure ZIF-8, Cy5.5@ZIF-8, and DOX@ZIF-8 NPs were also performed, and the corresponding results are shown in Figures S3–S5, which were highly consistent with the trends described above. In order to prove the dextran modification on the surfaces of these NPs, the energy-dispersive spectrometry (EDS) elemental mapping of ZIF-8-Dex NPs was performed, as well as the high-angle annular dark-field (HAADF) scanning transmission electron microscopy (STEM). As shown in Figure S6, the dextran modification on the surfaces of the ZIF-8-Dex NPs was evident from the results of EDS elemental mapping, where the presence of oxygen element (O) verified the surface decoration to some extent. This consequence is ascribed to the fact that the oxygen element only stemmed from the dextran, while it was absent from the other precursors of ZIF-8, as shown in Figure S7a,b. However, both of the loaded functional molecules contain oxygen (Figure S7c,d) and, thus, we investigated the EDS elemental mapping of ZIF-8-Dex rather than Cy5.5&DOX@ZIF-8-Dex NPs. On the other hand, the as-prepared Cy5.5&DOX@ZIF-8-Dex NPs were able to retain the colloidal stability in various mediums, including phosphate-buffered saline (PBS), Roswell Park Memorial Institute-1640 (RPMI-1640), and fetal bovine serum (FBS), which was confirmed from the results of colloidal stability in Figure S8.

### 3.2. Characterization of the Cy5.5&DOX@ZIF-8-Dex NPs

With the successful preparation of the Cy5.5&DOX@ZIF-8-Dex NPs having been confirmed, the structure and performances of these composite NPs were characterized in further detail. The fluorescent spectra of Cy5.5&DOX@ZIF-8-Dex NPs exhibited a signal at 710 nm (Figure S2d), which was absent from the spectrum of the ZIF-8-Dex NPs, thus indicating that the Cy5.5 dyes were successfully loaded into the composite NPs [33,34,35,36]. Moreover, the fluorescence intensities observed in the NIR region exhibited a positive correlation with the concentrations of Cy5.5&DOX@ZIF-8-Dex in the range from 50 to 200 μg/mL, which revealed that Cy5.5&DOX@ZIF-8-Dex could serve as an effective NIRF imaging agent. UV-Vis absorption spectra of free Cy5.5, free DOX, and Cy5.5&DOX@ZIF-8-Dex NPs (Figure S2e,f) were recorded to quantitatively evaluate the loading efficiencies of the functional molecules. Based on these measurements, the loading efficiencies for Cy5.5 and DOX were, respectively, calculated to be 3.5 ± 0.9% and 13.8 ± 2.9%. As was the case with the Cy5.5@ZIF-8-Dex and DOX@ZIF-8-Dex NPs, the spectra of the Cy5.5&DOX@ZIF-8-Dex NPs revealed that the peaks corresponding to the encapsulated Cy5.5 and DOX also exhibited red shifts. These findings thus confirmed that two distinct multifunctional molecules were successfully co-loaded within ZIF-8, thus establishing the foundation for the application of these composite NPs as a drug delivery system and pH-responsive release platforms.

**Figure 2:**
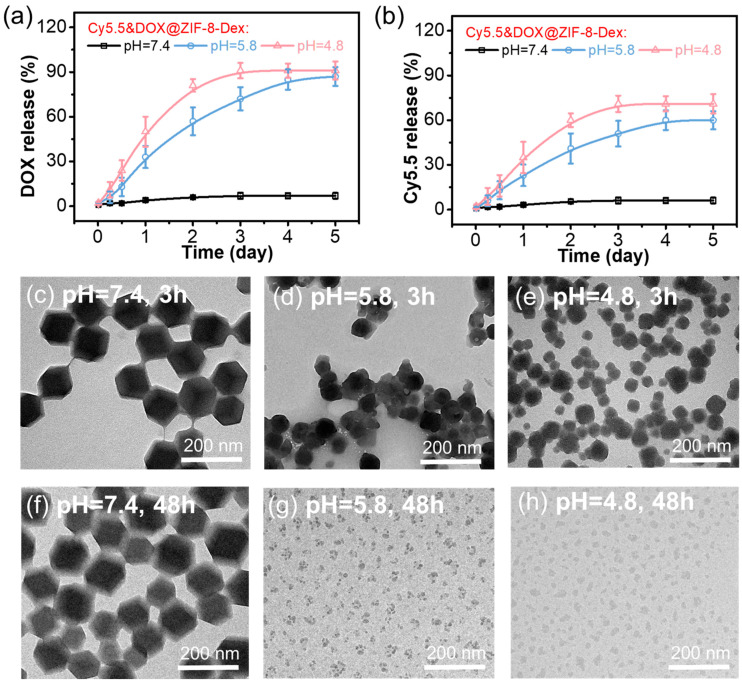
(**a**) DOX release from Cy5.5&DOX@ZIF-8-Dex at different pH values. (**b**) Cy5.5 release from Cy5.5&DOX@ZIF-8-Dex at various pH values. TEM images of Cy5.5&DOX@ZIF-8-Dex under different pH values for 3 h: (**c**) pH = 7.4, (**d**) pH = 5.8, and (**e**) pH = 4.8. TEM images of Cy5.5&DOX@ZIF-8-Dex under different pH values for 48 h: (**f**) pH = 7.4, (**g**) pH = 5.8, and (**h**) pH = 4.8.

The pH sensitivity of Cy5.5&DOX@ZIF-8-Dex NPs, resulting from the disassembly of the ZIF-8 structure under acidic environments [14,20], was evaluated by measuring the Cy5.5 and DOX release in buffer solutions with various pH values. As anticipated, the leakage rates of Cy5.5 and DOX from Cy5.5&DOX@ZIF-8-Dex at pH = 7.4 were nearly negligible (Figure S9c). However, the Cy5.5 and DOX release rates increased dramatically in response to reductions of the pH value (Figure 2a,b). This result was attributed to the disassembly of the ZIF-8 structure under acidic environments, which was confirmed by the TEM images of Cy5.5&DOX@ZIF-8-Dex under acidic conditions for two days (Figure 2c–h). In particular, the cumulative percentage of DOX release reached up to approximately 90% in buffer solution (pH = 4.8) after five days. Meanwhile, the cumulative percentage of Cy5.5 release in the same buffer solution after five days was only around 70%, which can be attributed to the hydrophobicity of Cy5.5. In particular, the released Cy5.5 might undergo partial aggregation with disassembled segments of ZIF-8 or other Cy5.5 molecules, leading to the retention of these aggregates during dialysis and an inefficient release of Cy5.5. Even in this situation, the remarkable pH-responsive release of DOX from the above discussion revealed that the Cy5.5&DOX@ZIF-8-Dex NPs would provide ideal nanocarriers for NIRF imaging and drug delivery applications. Additionally, the pH sensitivity of Cy5.5@ZIF-8-Dex and DOX@ZIF-8-Dex NPs were also performed in a similar way (Figure S9a,b), further confirming the pH-responsive behavior of ZIF-8 structure under acidic environments.

**Figure 3:**
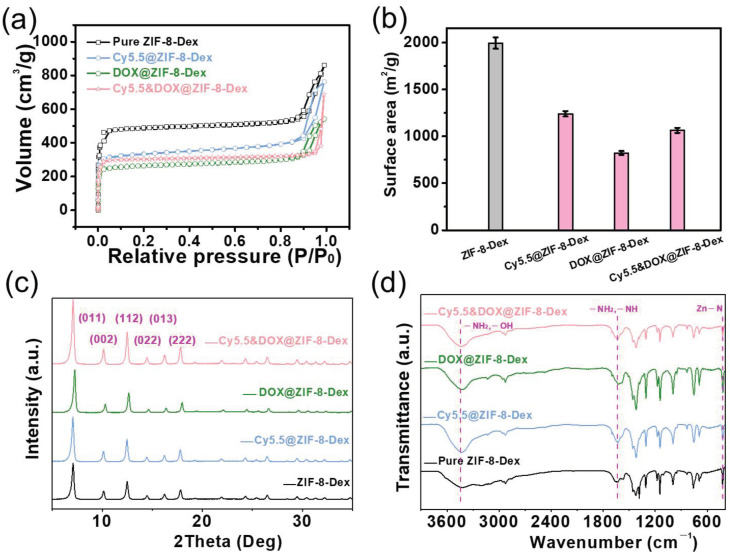
(**a**) Nitrogen sorption isotherms of various functional molecule@ZIF-8-Dex composite NPs. (**b**) Surface areas of various functional molecule@ZIF-8-Dex composite NPs. (**c**) XRD patterns of various functional molecule@ZIF-8-Dex composite NPs. (**d**) FTIR spectra of various functional molecule@ZIF-8-Dex composite NPs.

After the encapsulation and release of functional molecules were systematically investigated, the structural properties of Cy5.5&DOX@ZIF-8-Dex, including porosity and crystallinity, were subsequently characterized to explore their influence on the drug-loading capabilities of the ZIF-8-Dex NPs. Initially, the N_2_ adsorption–desorption curves of pure ZIF-8-Dex, Cy5.5@ZIF-8-Dex, DOX@ZIF-8-Dex, and Cy5.5&DOX@ZIF-8-Dex NPs at 77 K (Figure 3a) followed the type I isotherm [46,47]. The Brunauer–Emmett–Teller (BET) surface areas of the various ZIF-8-Dex systems revealed that the pure ZIF-8-Dex NPs possessed the highest surface area, and the surface areas decreased due to the encapsulation of functional molecules (Figure 3b). This trend suggested that the ZIF-8 content in the composite NPs had a direct influence on their porosities and surface areas. In other words, the encapsulation of functional molecules led to a reduction in the ZIF-8 content within the composite NPs, thus causing the effective surface area to decrease. The XRD patterns (Figure 3c) of all the composite NPs closely matched that of pure ZIF-8, which suggested that they had identical crystal structures in each case. In particular, all of the composite NPs exhibited similar characteristic peaks to those of pure ZIF-8 at angles of 7.2°, 10.4°, 12.8°, 14.5°, 16.3°, and 18.7°, which, respectively, corresponded to (011), (002), (112), (022), (013), and (222) crystal planes [30,48]. Moreover, the FTIR spectra of the various composite ZIF-8-Dex NPs were also very similar (Figure 3d), which provided further evidence that they possessed the same ZIF-8 structure regardless of whether they had encapsulated functional molecules. The characteristic peak at ~425 cm^−1^ was ascribed to the stretching vibrations of Zn-*n*, obviously demonstrating the formation of a ZIF-8 structure [14,17]. Moreover, the wide peak from 3300 to 3700 cm^−1^ was attributed to the stretching vibrations from dextran (O-H, -NH_2_) and ZIF-8 (-NH). As shown in Figure S10, the peak at 1550–1650 cm^−1^ from Cy5.5&DOX@ZIF-8 NPs was owing to the secondary amino group (-NH) from the 2-mim in ZIF-8 structure, while the appearance of a relatively stronger peak from Cy5.5&DOX@ZIF-8-Dex NPs in the same region resulted from the primary amino group (-NH_2_) from dextran, which further proves the surface decoration of dextran on these composite NPs. All of these results confirmed the integrity of the ZIF-8 structure in Cy5.5&DOX@ZIF-8-Dex NPs, which was the foundation for the porosity and pH sensitivity of these materials and thus laid the groundwork for high drug-loading and pH-responsive drug-release capabilities.

### 3.3. Cytotoxicity and Cellular Uptake

**Figure 4:**
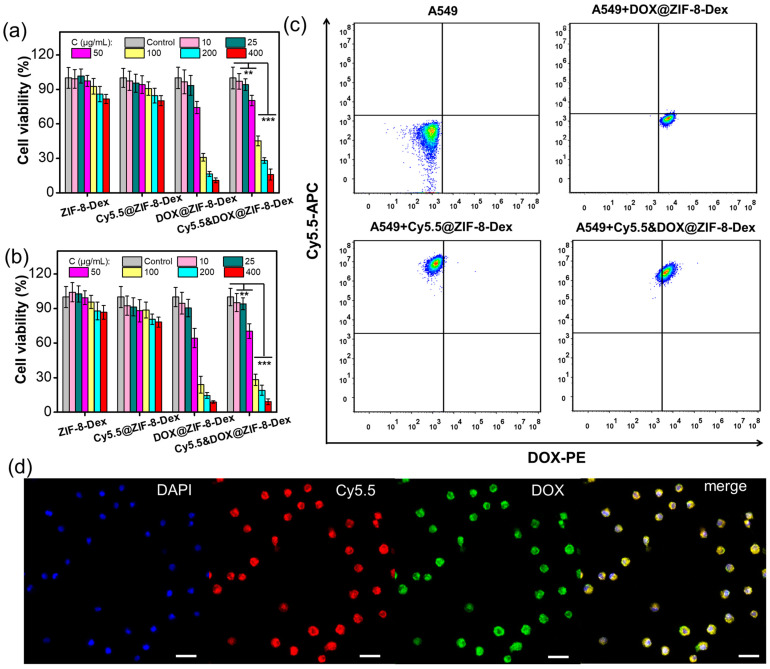
(**a**) Cell viabilities of A549 cells incubated with various ZIF-8-Dex composite NPs at various concentrations for 24 h (*n* = 6, ** *p* < 0.01, and *** *p* < 0.001). (**b**) Cell viability of A549 cells incubated with various ZIF-8-Dex composite NPs at various concentrations for 48 h (*n* = 6, ** *p* < 0.01, and *** *p* < 0.001). (**c**) Cell sorting analysis of A549 cells that were, respectively, co-incubated with DOX@XIF-8-Dex, Cy5.5@ZIF-8-Dex, and Cy5.5&DOX@ZIF-8-Dex NPs. (**d**) Confocal microscopy images of A549 cells incubated with Cy5.5&DOX@ZIF-8-Dex NPs for 3 h. The scale bars shown in all images correspond to 10 μm.

Regarding the considerable loading of DOX within the ZIF-8 structure, we further explored the feasibility of the as-prepared composite NPs as drug delivery systems for anticancer therapy. The cytotoxicities of Cy5.5&DOX@ZIF-8-Dex toward human umbilical vein endothelial cells (HUVEC) and human non-small-cell lung carcinoma cells (A549) were thus studied with the use of CCK-8 assays. As depicted in Figure 4a,b and Figure S11, it was found that the pure ZIF-8 and Cy5.5@ZIF-8-Dex NPs were negligibly toxic toward the HUVEC and A549 cells within the studied concentration range, thus implying the low cytotoxicity of the composite NPs in the absence of an anticancer drug. In contrast, the DOX@ZIF-8-Dex and Cy5.5&DOX@ZIF-8-Dex samples demonstrated concentration-dependent therapeutic effects on HUVEC and A549 cells due to the encapsulation of DOX within these composite NPs. In particular, the cell viabilities observed in the Cy5.5&DOX@ZIF-8-Dex treatment group were approximately 15.8% for 24 h and 9.1% for 48 h at the concentration of 400 μg/mL, respectively, indicating significant differences in comparison with control group (*n* = 6, *p* < 0.001).

The cellular uptake efficiency of Cy5.5&DOX@ZIF-8-Dex into A549 cells was evaluated via flow cytometry analysis. In comparison with the control group, the results observed for the phycoerythrin (PE) and allophycocyanin (APC) pathways in Cy5.5@ZIF-8-Dex, DOX@ZIF-8-Dex, and Cy5.5&DOX@ZIF-8-Dex confirmed that efficient cellular uptake was achieved (Figure 4c). Moreover, the confocal microscopy images of A549 cells that had received various treatments were accordingly obtained to observe the in vitro cellular uptake behavior of Cy5.5&DOX@ZIF-8-Dex with A549 cells. The cell nuclei were stained with DAPI and thus appeared blue, while the cytoplasm appeared red and green due to the existence of Cy5.5 and DOX, respectively. After the A549 cells were incubated with Cy5.5&DOX@ZIF-8-Dex, both red and green fluorescence signals were observed in Figure 4d, thus indicating that Cy5.5&DOX@ZIF-8-Dex NPs was internalized by the cells. Similarly, the CLSM images of the Cy5.5@ZIF-8-Dex and DOX@ZIF-8-Dex NPs showed that they had been taken up by the A549 cells (Figure S12, SI). Taken together, these findings confirmed the efficient cellular uptake performances of the composite NPs and thus demonstrated that they were promising candidates for in vivo applications in tumor theranostics.

### 3.4. In Vivo NIR Fluorescence Imaging

**Figure 5:**
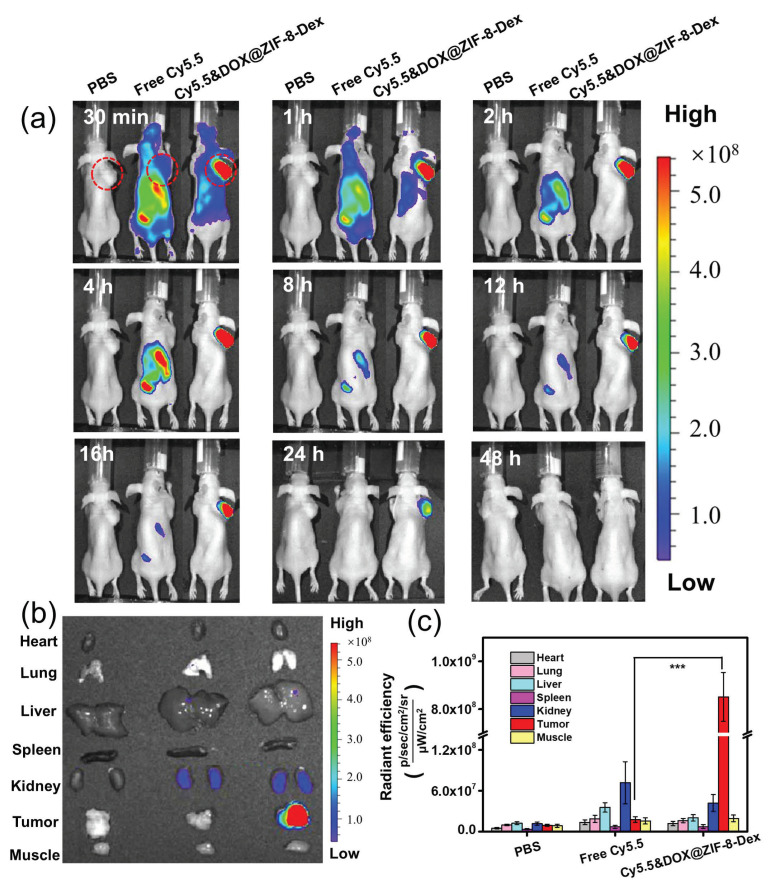
(**a**) In vivo fluorescent images of free Cy5.5 and Cy5.5&DOX@ZIF-8-Dex NPs in A549 tumor-bearing nude mice. The tumors are shown in red circles at the anterior armpits. (**b**) In vivo fluorescent images of organs harvested from A549 tumor-bearing nude mice that were, respectively, injected with PBS, free Cy5.5, and Cy5.5&DOX@ZIF-8-Dex NPs. (**c**) Fluorescence intensities observed in the organs shown in (**b**) (*n* = 6, *** *p* < 0.001).

Inspired by the desirable fluorescence emission in the NIR region, we further investigated the utilization of the Cy5.5&DOX@ZIF-8-Dex NPs for in vivo tumor fluorescence imaging. A549 tumor-bearing BALB/c mice were chosen as the animal models for these experiments (Figure S13). The A549 tumor cells were subcutaneously injected into the anterior armpits of these mice and the tumors were allowed to grow to a volume of ~150 mm^3^ prior to the in vivo fluorescence imaging observations. Subsequently, the A549 tumor-bearing models were intravenously injected with PBS buffer, free Cy5.5, and Cy5.5&DOX@ZIF-8-Dex, respectively. The NIRF images of each group were obtained with the use of the in vivo imaging system. It was evident that free Cy5.5 had circulated throughout the entire body during the initial stage and had subsequently accumulated in the liver and kidneys, without accumulating to significant degrees at the tumor sites (Figure 5a). Conversely, strong fluorescence signals were observed in the tumors of the A549 tumor-bearing mice that had been injected with Cy5.5&DOX@ZIF-8-Dex NPs, indicating that these NPs had accumulated at the tumor sites. In comparison, the fluorescence signals of A549 tumors in mice that had received the injection of Cy5.5&DOX@ZIF-8-Dex NPs were much stronger than those of the mice treated with free Cy5.5, thus demonstrating that Cy5.5&DOX@ZIF-8-Dex had readily accumulated at tumor sites due to the EPR effect [39,44]. The tumor sites exhibited the strongest fluorescence intensities during the period between 4 and 8 h following injection with the Cy5.5&DOX@ZIF-8-Dex NPs. Subsequently, the fluorescence intensities displayed by the metabolic organs in mice belonging to the free Cy5.5 treatment group and by the tumor sites of mice belonging to the Cy5.5&DOX@ZIF-8-Dex treatment group gradually decreased in both cases and had almost disappeared after 48 h post-injection. These observations were likely due to the deconstruction of the ZIF-8 structure and the metabolic degradation of free Cy5.5.

The fluorescent images of organs and tumors that were dissected from mice in the free Cy5.5 group and Cy5.5&DOX@ZIF-8-Dex NPs group are shown in Figure 5b, and stronger fluorescence was observed in the tumors collected from the Cy5.5&DOX@ZIF-8-Dex group than in those obtained from the free Cy5.5 group. In particular, the fluorescence intensity exhibited by a tumor harvested from a mouse treated with Cy5.5&DOX@ZIF-8-Dex was nearly 48 times stronger than that exhibited by a corresponding tumor collected from a mouse treated with free Cy5.5 (Figure 5c, *n* = 6, *p* < 0.001). Meanwhile, the metabolic organs (namely, the liver and kidneys) collected from mice in the free Cy5.5 and Cy5.5&DOX@ZIF-8-Dex treatment groups also exhibited strong fluorescence, which can be attributed to the metabolism of Cy5.5 dyes by these organs. These findings indicated that the Cy5.5&DOX@ZIF-8-Dex NPs are promising platforms for the NIR fluorescent imaging of tumors due to their tumor-targeted capabilities and the strong fluorescence in biological systems.

### 3.5. In Vivo Chemotherapy Effect

**Figure 6:**
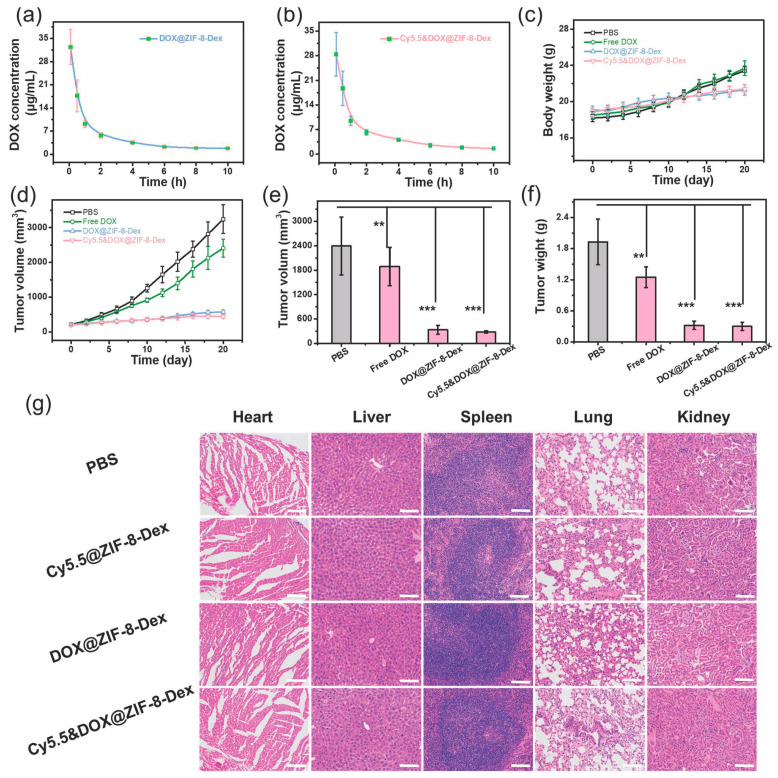
(**a**) Pharmacokinetic behavior of the DOX@ZIF-8 NPs in normal mice. (**b**) Pharmacokinetic behavior of the Cy5.5&DOX@ZIF-8 NPs in normal mice. (**c**) Changes in body weights of A549 tumor-bearing mice measured during a three-week period following various treatments. (**d**) Tumor growth curves of A549 tumor-bearing mice injected with PBS, free DOX, DOX@ZIF-8-Dex, and Cy5.5&DOX@ZIF-8-Dex NPs, respectively. (**e**) Dissected A549 tumor volumes after treatments (** *p* < 0.01, and *** *p* < 0.001). (**f**) Dissected A549 tumor weights after treatments (** *p* < 0.01, and *** *p* < 0.001). (**g**) H&E staining images of heart, liver, spleen, lung, and kidneys in A549 tumor-bearing mice. The scale bars correspond to 100 μm.

The pharmacokinetic behavior of DOX@ZIF-8-Dex and Cy5.5&DOX@ZIF-8-Dex NPs in A549 tumor-bearing mice was investigated prior to the chemotherapy evaluation. As shown in Figure 6a,b, the concentration of Cy5.5&DOX@ZIF-8-Dex in the blood decreased with the passage of time following injection due to the efficient accumulation of Cy5.5&DOX@ZIF-8-Dex NPs at tumor sites. The biosafety of all groups was further evaluated based on body weight measurements. None of the mice in the various treatment groups exhibited weight loss, indicating that the toxicity of the nanomaterials employed in this study were negligible (Figure 6c). The tumor volumes measured in mice during a three-week period after these mice had received various treatments are shown in Figure 6d. As expected, rapid tumor growth was observed in the PBS and free DOX treatment groups, while the DOX@ZIF-8-Dex and Cy5.5&DOX@ZIF-8-Dex treatment groups showed significant inhibition of tumor growth. The tumor growth rate of the free DOX group was not significantly suppressed, thus suggesting DOX had not accumulated to a sufficient degree at the tumor sites due to its relatively short circulation time. In contrast, the tumor growth rates of the DOX@ZIF-8-Dex and Cy5.5&DOX@ZIF-8-Dex treatment groups were extremely inhibited due to their tumor-targeting abilities thanks to the EPR effect [39,44].

Furthermore, photographs of the dissected tumors from the mice receiving different treatments are shown in Figure S14. It was indicated that only the DOX@ZIF-8-Dex and Cy5.5&DOX@ZIF-8-Dex treatment groups showed the desirable chemotherapeutical effects due to the sufficient accumulations of DOX at tumor sites. Furthermore, the volumes and weights of these dissected tumors were, respectively, measured and calculated (Figure 6e,f). The volumes and weights of the dissected tumors further demonstrated that the Cy5.5&DOX@ZIF-8-Dex NPs offered significant chemotherapeutical effects, which was in close agreement with the abovementioned results. In addition, histological analysis of each group was performed to further study the toxicity of the various composite NPs. The morphologies of these sectioned organs obtained from mice receiving different treatments were illustrated with the use of H&E staining in Figure 6g. The cellular integrity of the organs obtained from the mice in the various treatment groups did not significantly differ from those in the PBS treatment group. Taken together, our findings demonstrated that the Cy5.5&DOX@ZIF-8-Dex NPs have a significant potential as safe nano-systems for in vivo tumor theranostics applications.

## 4. Conclusions

In summary, uniform Cy5.5&DOX@ZIF-8 nanoparticles were efficiently synthesized using a one-pot method in a DMSO/H_2_O solvent mixture, allowing simultaneous encapsulation of hydrophilic DOX and hydrophobic Cy5.5 in a single step. The method enabled construction of the ZIF-8 architecture and encapsulation of functional molecules concurrently. Surface modification with amino-dextran enhanced the colloidal stability and biocompatibility. This approach effectively facilitated co-loading of chemotherapeutic drugs and imaging agents. Combined Cy5.5 and DOX molecules conferred a remarkable fluorescent imaging ability and pH-responsive chemotherapeutic effect to the composite NPs. In vivo NIRF imaging in A549 tumor-bearing mice demonstrated significant accumulation of Cy5.5 at tumor sites via the EPR effect, with fluorescence intensities approximately 48 times greater than those of free Cy5.5. Additionally, in vitro cytotoxicity assays revealed significant cytotoxicity against A549 tumor cells. In vivo chemotherapy with Cy5.5&DOX@ZIF-8-Dex NPs exhibited dramatically enhanced efficacy compared to free DOX, validating the tumor-targeting capability. These multifunctional ZIF-8-based nanocarriers advance tumor-targeting anticancer theranostics, expanding applications for acid-responsive NIR fluorescent imaging and chemotherapy in biomedicine.

## Data Availability

Data are contained within the article.

## Supplementary Materials

The following supporting information can be downloaded at: https://www.mdpi.com/article/10.3390/pharmaceutics16060823/s1, Figure S1. (a) UV-Vis absorption spectra of Cy5.5 at various concentrations. (b) UV-Vis absorption spectra of DOX at various concentrations. (c) Calibration curve for Cy5.5 at 675 nm. (d) Calibration curve for DOX at 488 nm. Figure S2. (a) UV-Vis absorption spectra of Cy5.5@ZIF-8 NPs prepared via one-pot and two-step methods. (b) UV-Vis absorption spectra of DOX@ZIF-8 NPs prepared via one-pot and two-step methods. (c) Drug loading efficiencies of Cy5.5@ZIF-8 and DOX@ZIF-8 composite NPs prepared via one-pot and two-step methods. (d) Fluorescence spectra of Cy5.5&DOX@ZIF-8-Dex composite NPs at various concentrations. The spectrum of pure ZIF-8 (recorded at 200 μg/mL) is shown for comparison. (e) UV-Vis absorption spectra of Cy5.5&DOX@ZIF-8-Dex NPs. (f) Drug loading efficiency of Cy5.5&DOX@ZIF-8-Dex. Figure S3. TEM images of various ZIF-8 NPs prepared via the one-pot methods: (a) The pure ZIF-8 NPs; (b) The Cy5.5@ZIF-8 composite NPs; and (c) The DOX@ZIF-8 composite NPs. Size distributions of various ZIF-8 NPs prepared via the one-pot method: (d) The pure ZIF-8 NPs; (e) The Cy5.5@ZIF-8 composite NPs; and (f) The DOX@ZIF-8 composite NPs. The scale bars shown in (a-c) correspond to 100 nm. Figure S4. (a) DLS curves of ZIF-8 NPs with or without dextran-modification. (b) DLS curves of Cy5.5@ZIF-8 NPs with or without dextran-modification. (c) DLS curves of DOX@ZIF-8 NPs with or without dextran-modification. Figure S5. (a) Zeta potentials of ZIF-8 NPs with or without dextran-modification. (b) Zeta potentials of Cy5.5@ZIF-8 NPs with or without dextran-modification. (c) Zeta potentials of DOX@ZIF-8 NPs with or without dextran-modification. Figure S6. (a) High-angle annular dark-field (HAADF) STEM images of dextran-modified ZIF-8-Dex NPs. (b–e) The energy-dispersive spectrometry (EDS) elemental mapping of various elements from the dextran-modified ZIF-8-Dex NPs. (f) The overlap results of various elements in EDS mapping from the dextran-modified ZIF-8-Dex NPs. The scale bars shown in all images correspond to 100 nm. Figure S7. (a) Molecular structure of dextran. (b) Molecular structure of 2-Methylimidazole that is main composition of ZIF-8. (c) Molecular structure of hydrophobic Cy5.5 dye. (d) Molecular structure of hydrophilic DOX. Figure S8. Colloidal stability of Cy5.5&DOX@ZIF-8-Dex nanoparticles dispersed in different mediums over the span of a week. Figure S9. (a) Cy5.5 release from Cy5.5@ZIF-8-Dex NPs at various pH values. (b) DOX release from DOX@ZIF-8-Dex NPs at different pH values. (c) Cy5.5 and DOX leakage rate of Cy5.5&DOX@ZIF-8-Dex at pH = 7.4. Figure S10. FTIR spectra of dextran, Cy5.5&DOX@ZIF-8 and Cy5.5&DOX@ZIF-8-Dex NPs. Figure S11. (a) Cell viabilities of HUVEC cells incubated with various ZIF-8-Dex composite NPs at various concentrations for 24 h (n = 6, * *p* < 0.05, ** *p* < 0.01, and *** *p* < 0.001). (b) Cell viability of HUVEC cells incubated with various ZIF-8-Dex composite NPs at various concentrations for 48 h (n = 6, * *p* < 0.05, ** *p* < 0.01, and *** *p* < 0.001). Figure S12. Confocal microscopy images of A549 cells which had been incubated with Cy5.5@ZIF-8-Dex and DOX@ZIF-8-Dex NPs for 3 h, respectively. The scale bars shown in all images correspond to 10 μm. Figure S13. Photographs of: (a) BALB/c nude mouse and (b) A549 tumor-bearing BALB/c mouse. The tumors were shown in red circle at the anterior armpit. Figure S14. Photographs of A549 tumors after the injection with PBS, DOX, DOX@ZIF-8-Dex and Cy5.5&DOX@ZIF-8-Dex NPs for three weeks, respectively.

## References

[1] Chen M., Dong R.H., Zhang J.J., Tang H., Li Q.Z., Shao H.W., Jiang X.Y. (2021). Nanoscale Metal–Organic Frameworks That Are Both Fluorescent and Hollow for Self-Indicating Drug Delivery. ACS Appl. Mater. Inter..

[2] Wang M., Zhang X., Chang Q., Zhang H., Zhang Z., Li K., Liu H., Liu D., An L., Tian Q. (2023). Tumor microenvironment–mediated NIR-I-to-NIR-II transformation of Au self-assembly for theranostics. Acta Biomater..

[3] Zheng X., Wang L., Guan Y., Pei Q., Jiang J., Xie Z. (2020). Integration of metal-organic framework with a photoactive porous-organic polymer for interface enhanced phototherapy. Biomaterials.

[4] Zhang Y., Wong C., Lim C.Z.J., Chen Q., Yu Z., Natalia A., Wang Z., Pang Q., Lim S.W., Loh T.P. (2023). Multiplexed RNA profiling by regenerative catalysis enables blood-based subtyping of brain tumors. Nat. Commun..

[5] Wang H., Yu D., Fang J., Cao C., Liu Z., Ren J., Qu X. (2019). Renal-Clearable Porphyrinic Metal-Organic Framework Nanodots for Enhanced Photodynamic Therapy. ACS Nano.

[6] Yang Y., Deng Y., Huang J., Fan X., Cheng C., Nie C., Ma L., Zhao W., Zhao C. (2019). Size-Transformable Metal-Organic Framework-Derived Nanocarbons for Localized Chemo-Photothermal Bacterial Ablation and Wound Disinfection. Adv. Funct. Mater..

[7] Corma A., García H., Llabrés i Xamena F.X. (2010). Engineering Metal Organic Frameworks for Heterogeneous Catalysis. Chem. Rev..

[8] Banerjee R., Phan A., Wang B., Knobler C., Furukawa H., O’Keeffe M., Yaghi O.M. (2008). High-Throughput Synthesis of Zeolitic Imidazolate Frameworks and Application to CO_2_ Capture. Science.

[9] Wang Z.Y., Wang C. (2021). Excited State Energy Transfer in Metal-Organic Frameworks. Adv. Mater..

[10] Yang G.L., Jiang X.L., Xu H., Zhao B. (2021). Applications of MOFs as Luminescent Sensors for Environmental Pollutants. Small.

[11] Chen Y., Lyu R., Wang J., Cheng Q., Yu Y., Yang S., Mao C., Yang M. (2023). Metal–organic frameworks nucleated by silk fibroin and modified with tumor-targeting peptides for targeted multimodal cancer therapy. Adv. Sci..

[12] Hayashi H., Côté A.P., Furukawa H., O’Keeffe M., Yaghi O.M. (2007). Zeolite A imidazolate frameworks. Nat. Mater..

[13] Ding H., Wei J., Fang L., Feng L., Gai S., He F., Wu L., Rehman Z., Yang P. (2024). A multichannel metabolic pathway interference strategy for complete energy depletion-mediated cancer therapy. Adv. Funct. Mater..

[14] Sun Q., Bi H., Wang Z., Li C., Wang X., Xu J., Zhu H., Zhao R., He F., Gai S. (2019). Hyaluronic acid-targeted and pH-responsive drug delivery system based on metal-organic frameworks for efficient antitumor therapy. Biomaterials.

[15] Zhao H., Shu G., Zhu J., Fu Y., Gu Z., Yang D. (2019). Persistent luminescent metal-organic frameworks with long-lasting near infrared emission for tumor site activated imaging and drug delivery. Biomaterials.

[16] Sun X., Liang X., Wang Y., Ma P., Xiong W., Qian S., Cui Y., Zhang H., Chen X., Tian F. (2023). A tumor microenvironment-activatable nanoplatform with phycocyanin-assisted in-situ nanoagent generation for synergistic treatment of colorectal cancer. Biomaterials.

[17] Lv Y., Ding D., Zhuang Y., Feng Y., Shi J., Zhang H., Zhou T., Chen H., Xie R. (2019). Chromium-Doped Zinc Gallogermanate@Zeolitic Imidazolate Framework-8: A Multifunctional Nanoplatform for Rechargeable In Vivo Persistent Luminescence Imaging and pH-Responsive Drug Release. ACS Appl. Mater. Inter..

[18] Zhang H., Li Q., Liu R., Zhang X., Li Z., Luan Y. (2018). A Versatile Prodrug Strategy to In Situ Encapsulate Drugs in MOF Nanocarriers: A Case of Cytarabine-IR820 Prodrug Encapsulated ZIF-8 toward Chemo-Photothermal Therapy. Adv. Funct. Mater..

[19] Wang T., Li S., Zou Z., Hai L., Yang X., Jia X., Zhang A., He D., He X., Wang K.A. (2018). zeolitic imidazolate framework-8-based indocyanine green theranostic agent for infrared fluorescence imaging and photothermal therapy. J. Mater. Chem. B.

[20] Chen H., Yang J., Sun L., Zhang H., Guo Y., Qu J., Jiang W., Chen W., Ji J., Yang Y. (2019). Synergistic Chemotherapy and Photodynamic Therapy of Endophthalmitis Mediated by Zeolitic Imidazolate Framework-Based Drug Delivery Systems. Small.

[21] Dong J., Chai X., Xue Y., Shen S., Chen Z., Wang Z., Yinwang E., Wang S., Chen L., Wu F. (2024). ZIF-8-Encapsulated Pexidartinib Delivery via Targeted Peptide-Modified M1 Macrophages Attenuates MDSC-Mediated Immunosuppression in Osteosarcoma. Small.

[22] Wang Q., Yu Y., Chang Y., Xu X., Wu M., Ediriweera G.R., Peng H., Zhen X., Jiang X., Searles D.J. (2023). Fluoropolymer-MOF Hybrids with Switchable Hydrophilicity for 19F MRI-Monitored Cancer Therapy. ACS Nano.

[23] Bhattacharjee S., Jang M.S., Kwon H.J., Ahn W.S. (2014). Zeolitic Imidazolate Frameworks: Synthesis, Functionalization, and Catalytic/Adsorption Applications. Catal. Surv. Asia.

[24] Shen J., Ma M., Zhang H., Yu H., Xue F., Hao N., Chen H. (2020). Microfluidics-Assisted Surface Tri-Functionalization of Zeolitic Imidazolate Framework Nanocarrier for Targeted and Controllable Multitherapies of Tumor. ACS Appl. Mater. Inter..

[25] Pan Y., Liu Y., Zeng G., Zhao L., Lai Z. (2011). Rapid synthesis of zeolitic imidazolate framework-8 (ZIF-8) nanocrystals in an aqueous system. Chem. Commun..

[26] Zheng H., Zhang Y., Liu L., Wan W., Guo P., Nyström A.M., Zou X. (2016). One-pot Synthesis of Metal–Organic Frameworks with Encapsulated Target Molecules and Their Applications for Controlled Drug Delivery. J. Am. Chem. Soc..

[27] Han Y., Zhao S., Wang F., Jiang J. (2023). In Situ Transformable Pro-nanotheranostic Platform for Activable Photoacoustic Imaging and Synergistic Photothermal/Chemodynamic Cancer Therapy. Anal. Chem..

[28] Zhang C., Hong S., Liu M.D., Yu W.Y., Zhang M.K., Zhang L., Zeng X., Zhang X.Z. (2020). pH-sensitive MOF integrated with glucose oxidase for glucose-responsive insulin delivery. J. Control. Release.

[29] Shu F., Lv D., Song X.L., Huang B., Wang C., Yu Y., Zhao S.C. (2018). Fabrication of a hyaluronic acid conjugated metal organic framework for targeted drug delivery and magnetic resonance imaging. RSC Adv..

[30] Wang X., Li X., Liang X., Liang J., Zhang C., Yang J., Wang C., Kong D., Sun H. (2018). ROS-responsive capsules engineered from green tea polyphenol–metal networks for anticancer drug delivery. J. Mater. Chem. B.

[31] Liang Z., Yang Z., Yuan H., Wang C., Qi J., Liu K., Cao R., Zheng H. (2018). A protein@metal–organic framework nanocomposite for pH-triggered anticancer drug delivery. Dalton Trans..

[32] Zhuang J., Kuo C.H., Chou L.Y., Liu D.Y., Weerapana E., Tsung C.K. (2014). Optimized Metal–Organic-Framework Nanospheres for Drug Delivery: Evaluation of Small-Molecule Encapsulation. ACS Nano.

[33] Guo H., Liu L., Hu Q., Dou H. (2022). Monodisperse ZIF-8@dextran Nanoparticles Co-loaded with Hydrophilic and Hydrophobic Functional Cargos for Combined Near-infrared Fluorescence Imaging and Photothermal Therapy. Acta Biomater..

[34] Guo H., Liu L., Hu Q., Dou H. (2021). Mixed Solvent Method for Improving the Size Uniformity and Cargo-Loading Efficiency of ZIF-8 Nanoparticles. Langmuir.

[35] Chen P., Kuang W., Zheng Z., Yang S., Liu Y., Su L., Zhao K., Liang G. (2019). Carboxylesterase-Cleavable Biotinylated Nanoparticle for Tumor-Dual Targeted Imaging. Theranostics.

[36] Liang X., Fang L., Li X., Zhang X., Wang F. (2017). Activatable near infrared dye conjugated hyaluronic acid-based nanoparticles as a targeted theranostic agent for enhanced fluorescence/CT/photoacoustic imaging guided photothermal therapy. Biomaterials.

[37] Li J., Li X., Gong S., Zhang C., Qian C., Qiao H., Sun M. (2020). Dual-mode avocado-like all-iron nanoplatform for enhanced T1/T2 MRI-guided cancer theranostic therapy. Nano Lett..

[38] Ma B., Zhuang W., He H., Su X., Yu T., Hu J., Yang L., Li G., Wang Y. (2019). Two-photon AIE probe conjugated theranostic nanoparticles for tumor bioimaging and pH-sensitive drug delivery. Nano Res..

[39] Duan X., Li Y. (2013). Physicochemical Characteristics of Nanoparticles Affect Circulation, Biodistribution, Cellular Internalization, and Trafficking. Small.

[40] Simone E.A., Dziubla T.D., Muzykantov V.R. (2008). Polymeric carriers: Role of geometry in drug delivery. Expert Opin. Drug Del..

[41] Huang W., Leng T., Gao M., Hu Q., Liu L., Dou H. (2020). Scalable dextran-polypyrrole nano-assemblies with photothermal/photoacoustic dual capabilities and enhanced biocompatibility. Carbohydr. Polym..

[42] Wang C., Guan W., Chen R., Levi-Kalisman Y., Xu Y., Zhang L., Zhou M., Xu G., Dou H. (2020). Fluorescent glycan nanoparticle-based FACS assays for the identification of genuine drug-resistant cancer cells with differentiation potential. Nano Res..

[43] Mukwaya V., Zhang P., Guo H., Dang-I A.Y., Hu Q., Li M., Mann S., Dou H. (2020). Fluorescent glycan nanoparticle-based FACS assays for the identification of genuine drug-resistant cancer cells with differentiation potential. Nano Res..

[44] Decuzzi P., Godin B., Tanaka T., Lee S.Y., Chiappini C., Liu X., Ferrari M. (2010). Size and shape effects in the biodistribution of intravascularly injected particles. J. Control. Release.

[45] Horcajada P., Chalati T., Serre C., Gillet B., Sebrie C., Baati T., Eubank J.F., Heurtaux D., Clayette P., Kreuz C. (2009). Porous metal–organic-framework nanoscale carriers as a potential platform for drug delivery and imaging. Nature Mater..

[46] Ortiz A.U., Freitas A.P., Boutin A., Fuchs A.H., Coudert F.X. (2014). What makes zeolitic imidazolate frameworks hydrophobic or hydrophilic? The impact of geometry and functionalization on water adsorption. Phys. Chem. Chem. Phys..

[47] Gan L., Chidambaram A., Fonquernie P.G., Light M.E., Choquesillo-Lazarte D., Huang H., Solano E., Fraile J., Viñas C., Teixidor F. (2020). A Highly Water-Stable meta-Carborane-Based Copper Metal–Organic Framework for Efficient High-Temperature Butanol Separation. J. Am. Chem. Soc..

[48] Qiao X., Su B., Liu C., Song Q., Luo D., Mo G., Wang T. (2017). Selective Surface Enhanced Raman Scattering for Quantitative Detection of Lung Cancer Biomarkers in Superparticle@MOF Structure. Adv. Mater..

