# Supplementary material for: Acid-Responsive Decomposable Nanomedicine Based on Zeolitic Imidazolate Frameworks for Near-Infrared Fluorescence Imaging/Chemotherapy Combined Tumor Theranostics
Source: Pharmaceutics. 2024 Jun 18;16(6):823. doi: 10.3390/pharmaceutics16060823 (PMC11207643; doi:10.3390/pharmaceutics16060823)
Supplement: Supplementary file 1 [file pharmaceutics-16-00823-s001.zip › pharmaceutics-3028032-supplementary.pdf]

Supporting Information

Acid-Responsive Decomposable Nanomedicine Based on Zeolitic Imidazolate Frameworks for Near-Infrared Fluorescence Imaging/Chemotherapy Combined Tumor Theranostics

Heze Guo, Vincent Mukwaya, Daikun Wu, Shuhan Xiong and Hongjing Dou *

The State Key Laboratory of Metal Matrix Composites, School of Materials Science and Engineering, Shanghai Jiao Tong University, Shanghai 200240, China

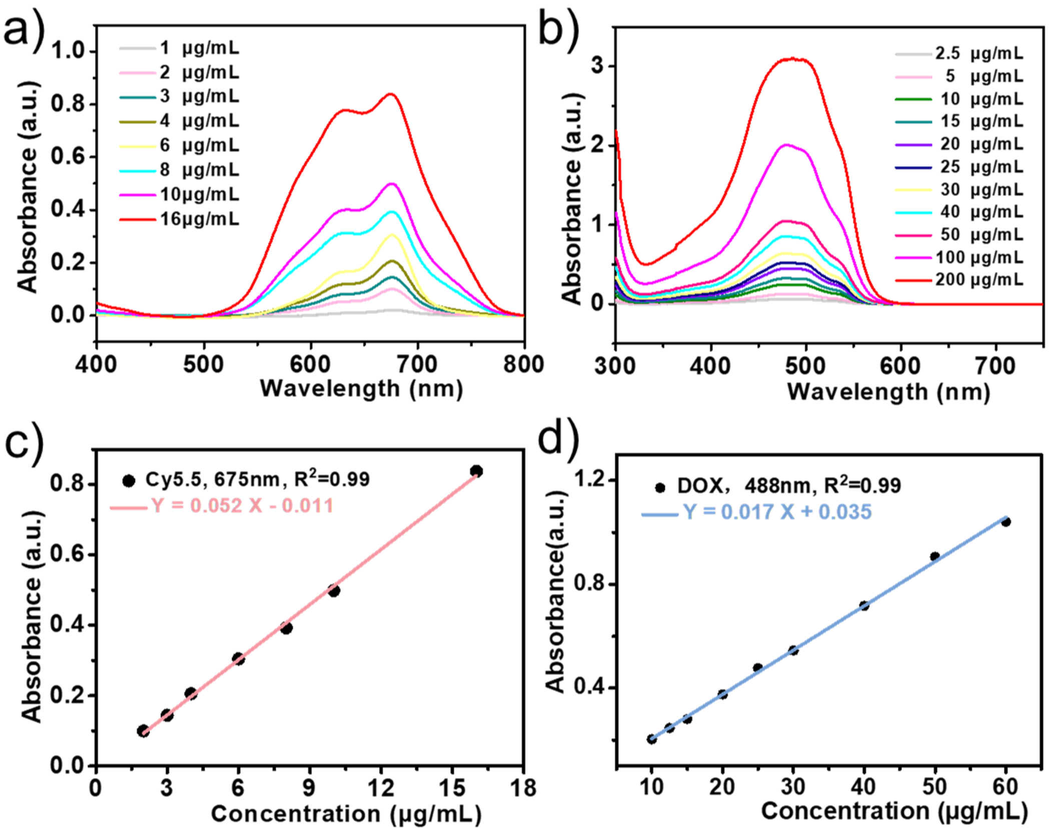

Figure S1. (a) UV-Vis absorption spectra of Cy5.5 at various concentrations. (b) UV-Vis absorption spectra of DOX at various concentrations. (c) Calibration curve for Cy5.5 at 675 nm. (d) Calibration curve for DOX at 488 nm.

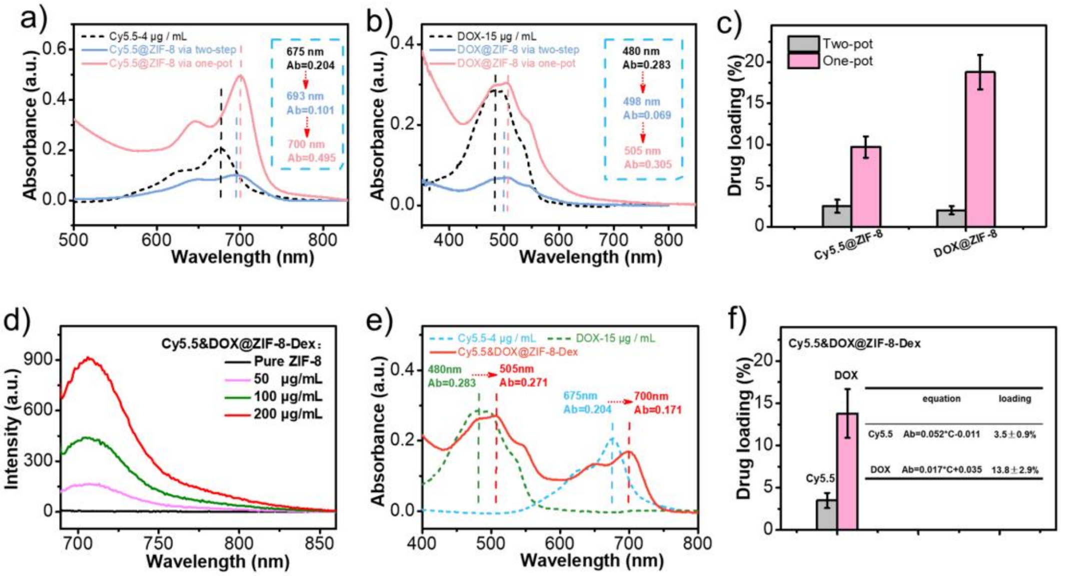

Figure S2. (a) UV-Vis absorption spectra of Cy5.5@ZIF-8 NPs prepared via one-pot and two-step methods. (b) UV-Vis absorption spectra of DOX@ZIF-8 NPs prepared via one-pot and two-step methods. (c) Drug loading efficiencies of Cy5.5@ZIF-8 and DOX@ZIF-8 composite NPs prepared via one-pot and two-step methods. (d) Fluorescence spectra of Cy5.5&DOX@ZIF-8-Dex composite NPs at various concentrations. The spectrum of pure ZIF-8 (recorded at 200 μg/mL) is shown for comparison. (e) UV-Vis absorption spectra of Cy5.5&DOX@ZIF-8-Dex NPs. (f) Drug loading efficiency of Cy5.5&DOX@ZIF-8-Dex.

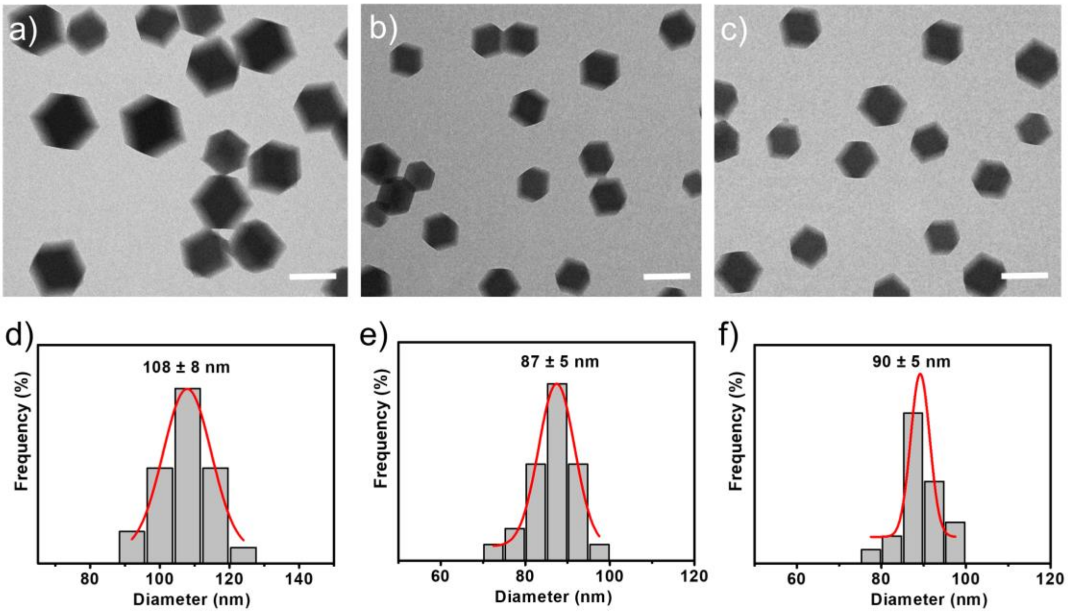

Figure S3. TEM images of various ZIF-8 NPs prepared via the one-pot methods: (a) The pure ZIF-8 NPs; (b) The Cy5.5@ZIF-8 composite NPs; and (c) The DOX@ZIF-8 composite NPs. Size distributions of various ZIF-8 NPs prepared via the one-pot method: (d) The pure ZIF-8 NPs; (e) The Cy5.5@ZIF-8 composite NPs; and (f) The DOX@ZIF-8 composite NPs. The scale bars shown in (a-c) correspond to 100 nm.

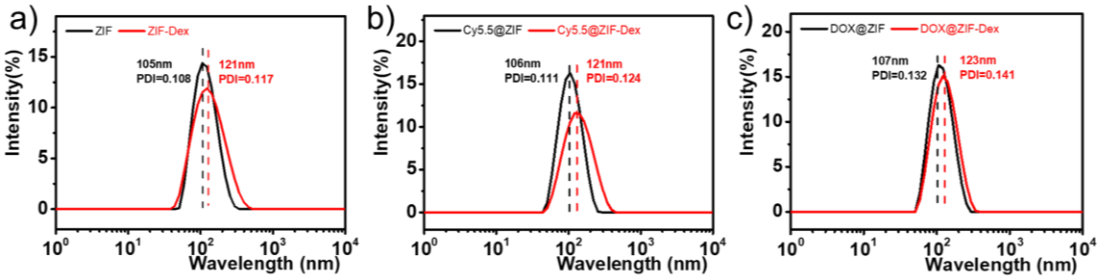

Figure S4. (a) DLS curves of ZIF-8 NPs with or without dextran-modification. (b) DLS curves of Cy5.5@ZIF-8 NPs with or without dextran-modification. (c) DLS curves of DOX@ZIF-8 NPs with or without dextran-modification.

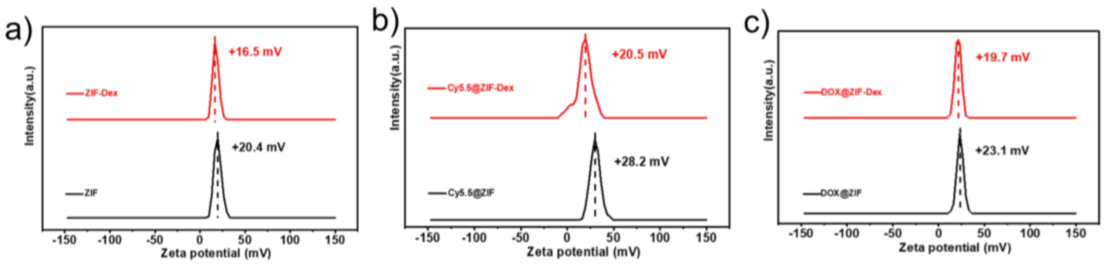

Figure S5. (a) Zeta potentials of ZIF-8 NPs with or without dextran-modification. (b) Zeta potentials of Cy5.5@ZIF-8 NPs with or without dextran-modification. (c) Zeta potentials of DOX@ZIF-8 NPs with or without dextran-modification.

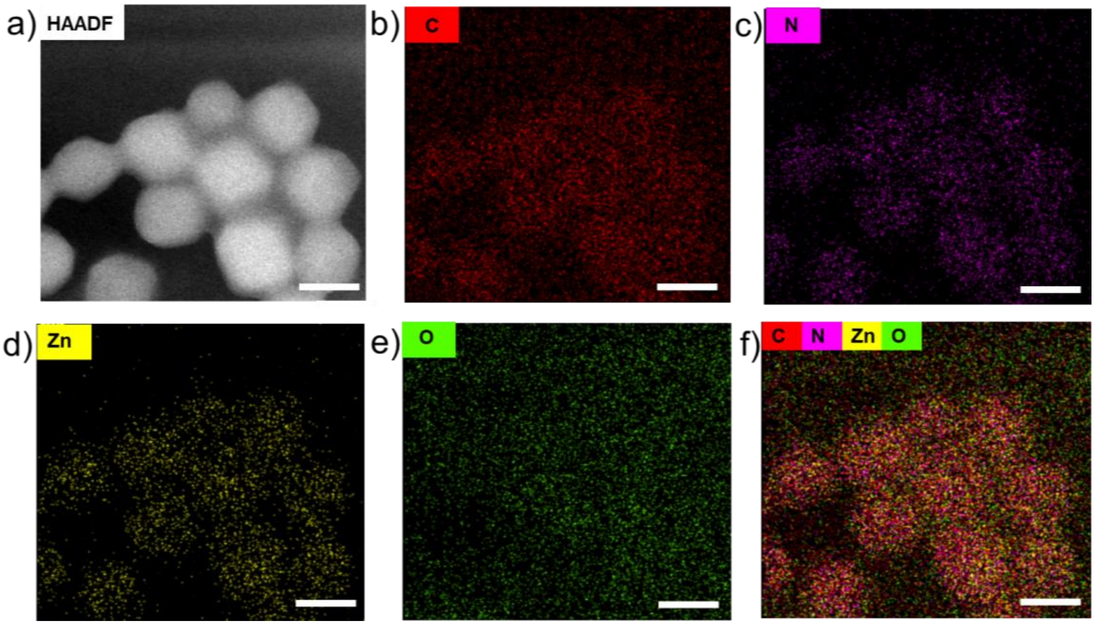

Figure S6. (a) High-angle annular dark-field (HAADF) STEM images of dextran-modified ZIF-8-Dex NPs. (b-e) The energy-dispersive spectrometry (EDS) elemental mapping of various elements from the dextran-modified ZIF-8-Dex NPs. (f) The overlap results of various elements in EDS mapping from the dextran-modified ZIF-8-Dex NPs. The scale bars shown in all images correspond to 100 nm.

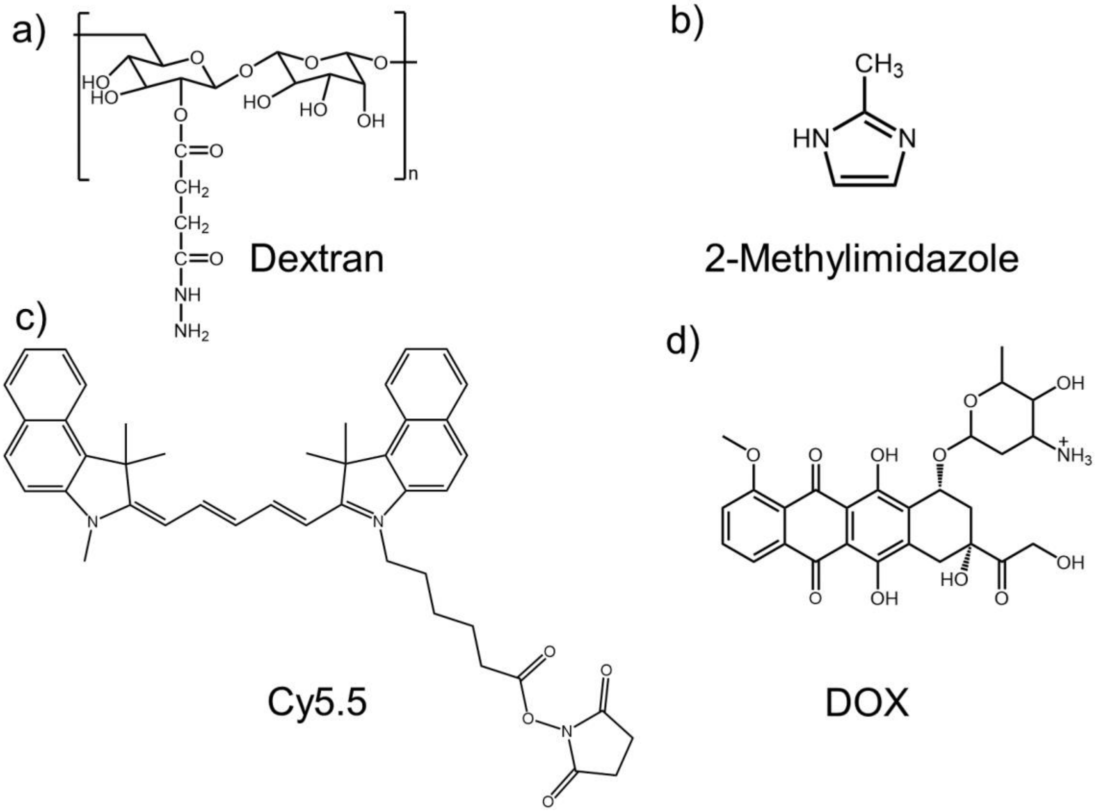

Figure S7. (a) Molecular structure of dextran. (b) Molecular structure of 2-Methylimidazole that is main composition of ZIF-8. (c) Molecular structure of hydrophobic Cy5.5 dye. (d) Molecular structure of hydrophilic DOX.

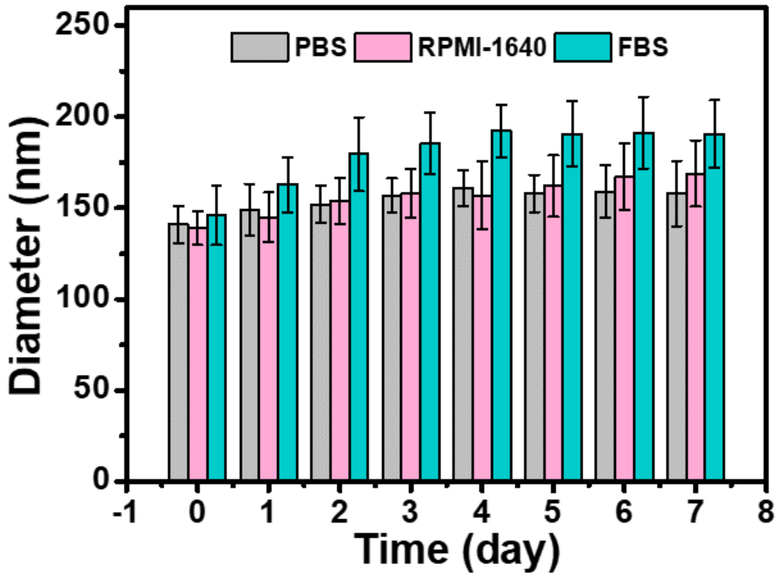

Figure S8. Colloidal stability of Cy5.5&DOX@ZIF-8-Dex nanoparticles dispersed in different mediums over the span of a week.

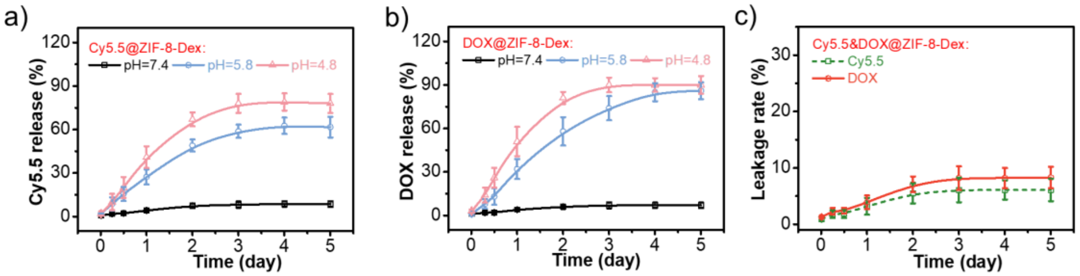

Figure S9. (a) Cy5.5 release from Cy5.5 @ZIF-8-Dex NPs at various pH values. (b) DOX release from DOX@ZIF-8-Dex NPs at different pH values. (c) Cy5.5 and DOX leakage rate of Cy5.5&DOX@ZIF-8-Dex at pH = 7.4.

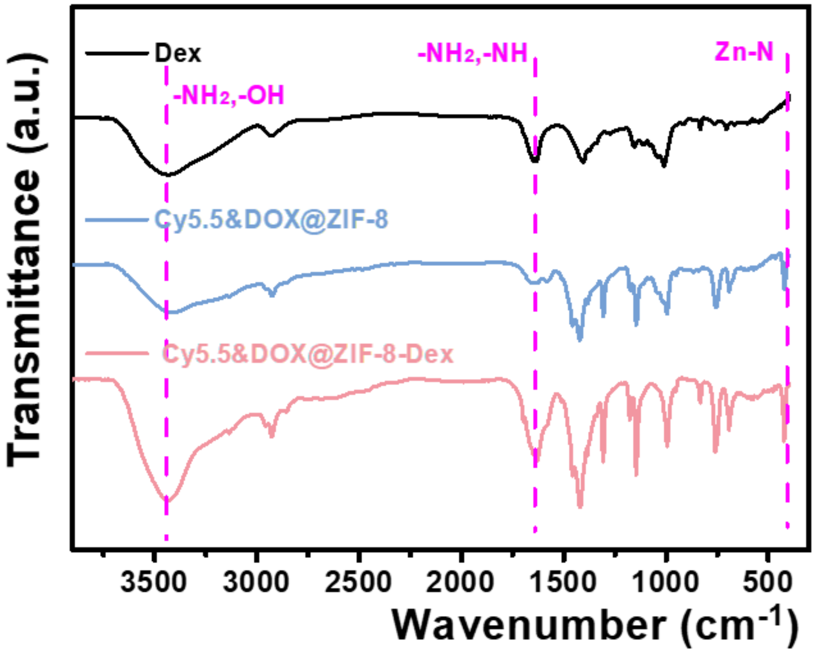

Figure S10. FTIR spectra of dextran, Cy5.5&DOX@ZIF-8 and Cy5.5&DOX@ZIF-8-Dex NPs.

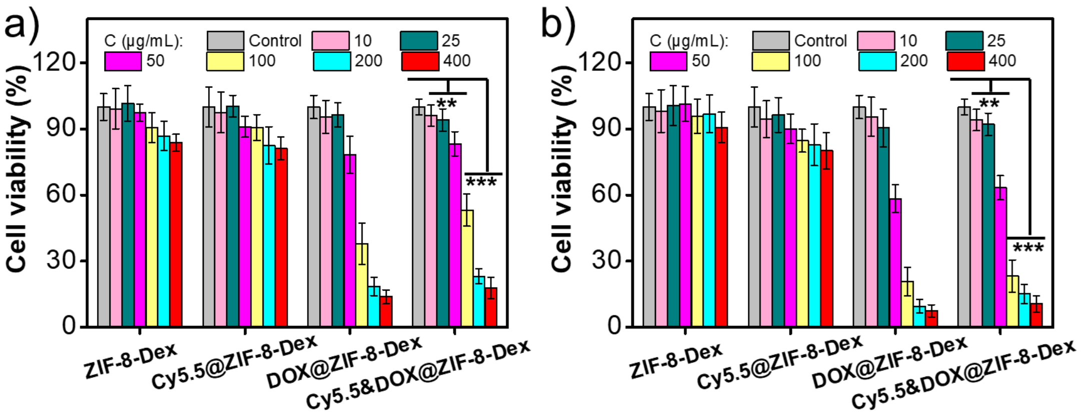

Figure S11. (a) Cell viabilities of HUVEC cells incubated with various ZIF-8-Dex composite NPs at vari-ous concentrations for 24 h (n = 6, $^{**}p < 0.01$, and $^{***}p < 0.001$). (b) Cell viability of HUVEC cells incubated with various ZIF-8-Dex composite NPs at various concentrations for 48 h (n = 6, $^{**}p < 0.01$, and $^{***}p < 0.001$).

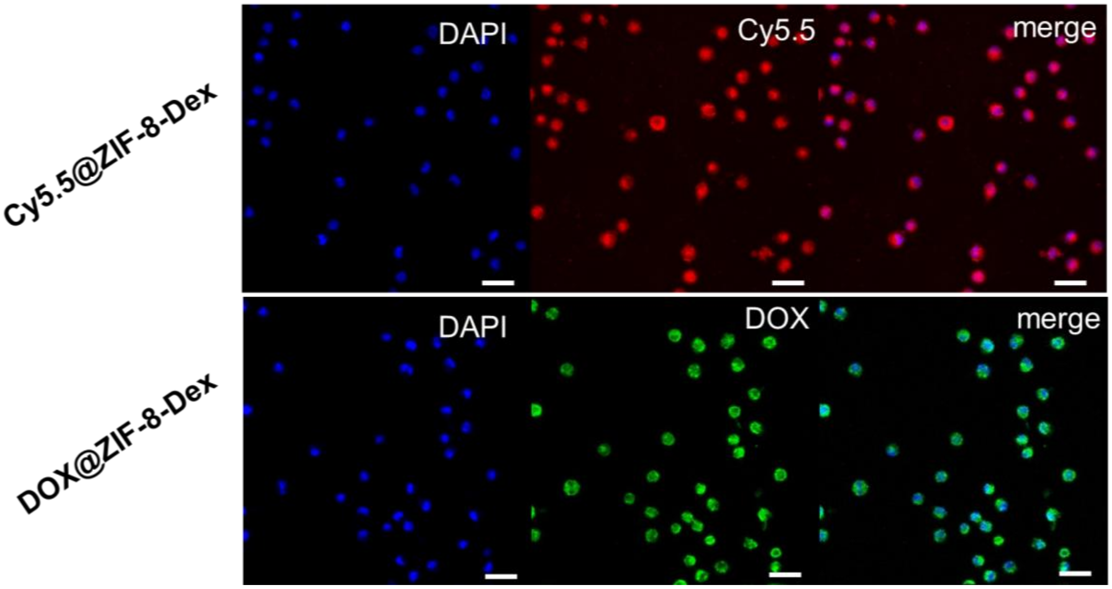

Figure S12. Confocal microscopy images of A549 cells which had been incubated with Cy5.5@ZIF-8-Dex and DOX@ZIF-8-Dex NPs for 3 h, respectively. The scale bars shown in all images correspond to 10 μm.

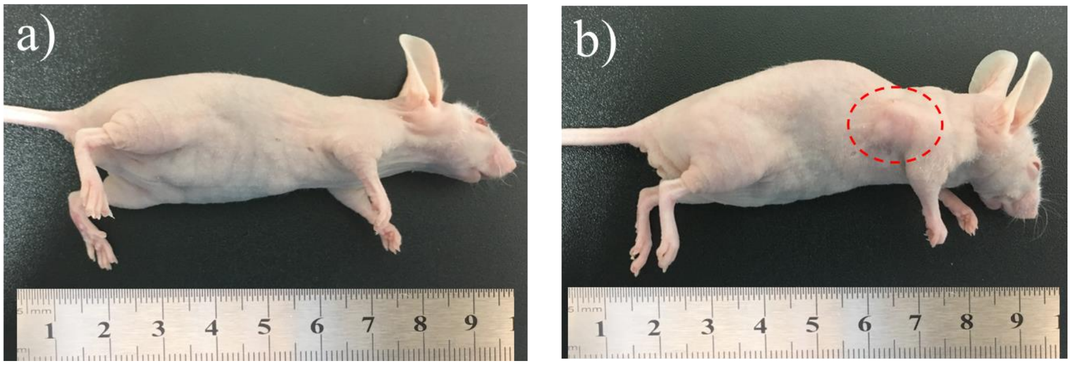

Figure S13. Photographs of: (a) BALB/c nude mouse and (b) A549 tumor-bearing BALB/c mouse. The tumors were shown in red circle at the anterior armpit.

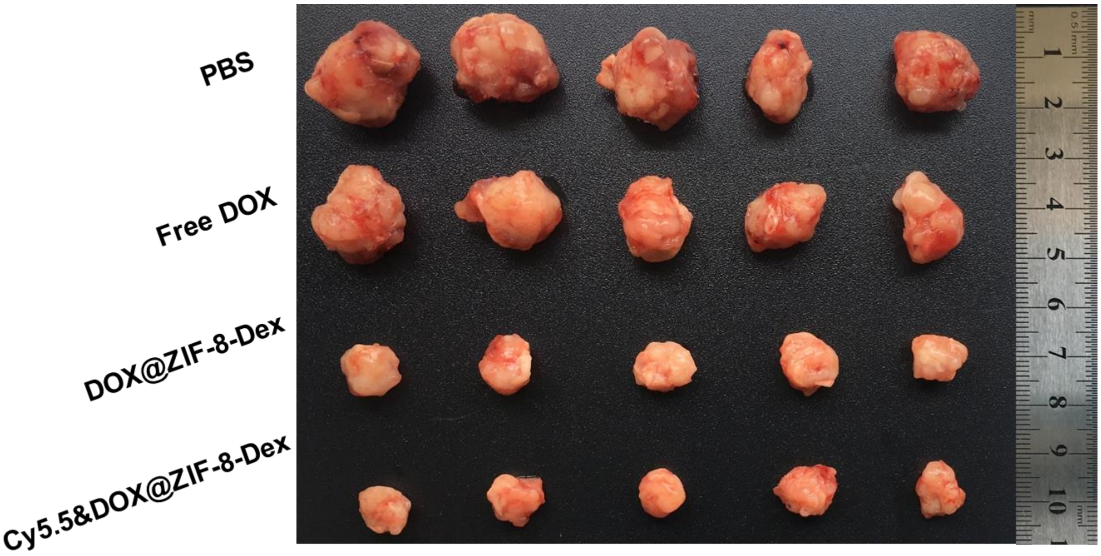

Figure S14. Photographs of A549 tumors after the injection with PBS, DOX, DOX@ZIF-8-Dex and Cy5.5&DOX@ZIF-8-Dex NPs for three weeks, respectively.
